# Research Progress on Solid-State Electrolytes in Solid-State Lithium Batteries: Classification, Ionic Conductive Mechanism, Interfacial Challenges

**DOI:** 10.3390/nano14221773

**Published:** 2024-11-05

**Authors:** Shun Ai, Xianli Wu, Jintao Wang, Xu Li, Xiaofeng Hao, Yuezhong Meng

**Affiliations:** 1 School of Materials Science and Engineering & College of Chemistry, Zhengzhou University, Zhengzhou 450001, China; 2 Institute of Chemistry, Henan Academy of Sciences, Zhengzhou 450046, China; 3 The Key Laboratory of Low-Carbon Chemistry & Energy Conservation of Guangdong Province, State Key Laboratory of Optoelectronic Materials and Technologies, School of Materials Science and Engineering, Sun Yat-sen University, Guangzhou 510275, China

**Keywords:** solid-state electrolytes, interfacial challenges, industrialization research status

## Abstract

Solid-state lithium batteries exhibit high-energy density and exceptional safety performance, thereby enabling an extended driving range for electric vehicles in the future. Solid-state electrolytes (SSEs) are the key materials in solid-state batteries that guarantee the safety performance of the battery. This review assesses the research progress on solid-state electrolytes, including polymers, inorganic compounds (oxides, sulfides, halides), and organic–inorganic composites, the challenges related to solid-state batteries in terms of their interfaces, and the status of industrialization research on solid-state electrolytes. For each kind of solid-state electrolytes, details on the preparation, properties, composition, ionic conductivity, ionic migration mechanism, and structure–activity relationship, are collected. For the challenges faced by solid-state batteries, the high interfacial resistance, the side reactions between solid-state electrolytes and electrodes, and interface instability, are mainly discussed. The current industrialization research status of various solid electrolytes is analyzed in regard to relevant enterprises from different countries. Finally, the potential development directions and prospects of high-energy density solid-state batteries are discussed. This review provides a comprehensive reference for SSE researchers and paves the way for innovative advancements in regard to solid-state lithium batteries.

## 1. Introduction

Lithium-ion batteries (LIBs) are integrated into our daily lives now and bring added convenience to travel and our daily lives [1]. It is important to improve the energy density, the duration, and the safety of batteries, to meet the demand for a new generation of batteries. As is well-known, traditional lithium-ion batteries involving graphite anode materials and liquid electrolytes have difficulty obtaining high-energy density, due to the theoretical limit related to the specific capacity (370 mAh/g) of graphite anodes [2,3], and present potential safety risks, due to the flammability and explosiveness of traditional organic liquid electrolytes (OLEs), and there is the potential for dendrite formation to puncture the separator, which may lead to short circuits at high currents [4,5]. In addition, OLEs are prone to oxidization and decomposition under a high voltage or high temperature, leading to the low cycling stability of batteries, which cannot meet the requirements of next-generation high-energy-density batteries. Solid-state lithium batteries with lithium metal as the anode materials and solid-state electrolytes (SSEs) as the ionic conductive medium can achieve high-energy density, due to the ultrahigh theoretical capacity (3860 mAh g^−1^) of lithium metal anodes and it having the lowest reduction potential of −3.04 V (vs. standard hydrogen electrodes) [6,7,8,9,10]. More importantly, SSEs have advantages over traditional liquid electrolytes, such as non-flammability, non-explosibility, and a wide electrochemical window, and they can inhibit the growth of lithium dendrites to improve the safety performance of lithium batteries [11,12], so they have been widely studied in recent years.

Solid-state electrolytes are the core materials in all solid-state lithium battery technology, largely determining the performance parameters of solid-state lithium batteries, such as the power density, cycle stability, safety, high and low temperatures, and service life. The ideal solid electrolyte should meet the following requirements [13]: (1) room temperature conductivity ≥10^−4^ mS cm^−1^; (2) electronic insulation (Li^+^ migration number is approximately 1); (3) wide electrochemical window (>5.5 V vs. Li/Li^+^); (4) good compatibility with electrode materials; (5) good thermal stability, wet environment resistance, excellent mechanical properties; (6) low-cost and easily obtained raw materials; and (7) a simple synthesis method. Researchers have made great efforts to design and prepare solid electrolytes that meet the relevant parameters and have made many breakthroughs. In the past two decades, many kinds of solid electrolytes with high ionic conductivity (σ_Li_^+^ > 1 mS cm^−1^) have been obtained and some of them even possess ultrahigh Li^+^ conductivities, surpassing conventional OLEs [14]. However, the industrial-scale application of solid-state electrolytes to lithium batteries still faces great challenges.

**Figure 1:**
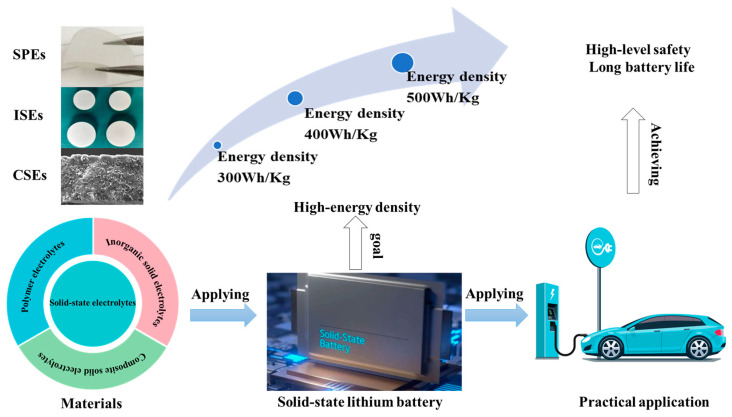
Framework for high-energy-density SSLBs: from materials research to practical application.

In this review, the preparation, composition, properties, ionic conductivity, and ionic transport mechanisms of solid polymer electrolytes, inorganic solid electrolytes (oxides, sulfides, halides), and organic–inorganic composite polymer electrolytes are introduced. The advantages and disadvantages of various solid-state electrolytes are summarized. The interface-related challenges between solid electrolytes and lithium metal anodes and cathodes, as well as the promising strategies for solving the interface-related issues are discussed in-depth. The current industrial application status of various solid-state electrolytes produced by relevant enterprises is collected. The structural design of solid-state electrolytes is proposed from the perspectives of preparation technology and the mechanism of the contact interface between solid-state electrolytes and electrodes, by using advanced machine learning and analysis, theoretical calculations and simulations, as guidance to solve the application problems related to solid electrolytes during industrialization and the mass production of solid batteries. In brief, this paper presents a basic and in-depth understanding of solid-state electrolytes made from electrolyte material and the challenges related to the use of solid-state electrolytes in SSLBs in industrial applications (shown as Figure 1).

## 2. The Progress on Solid-State Electrolytes

**Figure 2:**
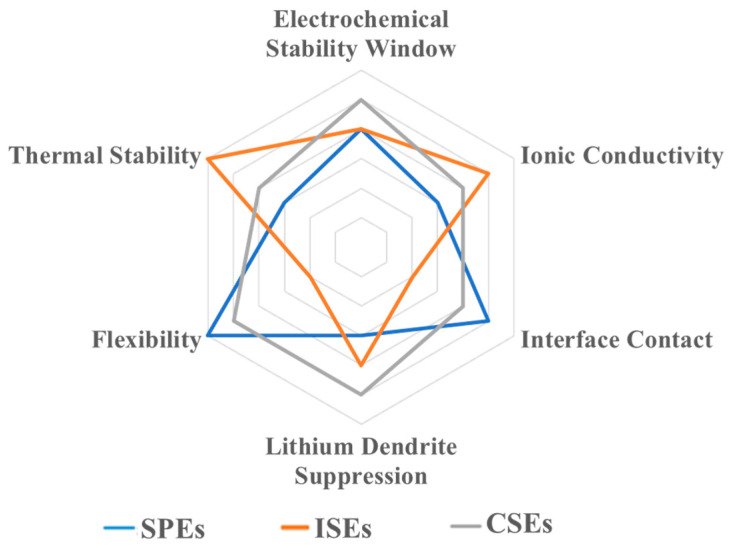
Property comparison of solid polymer electrolytes (SPEs), inorganic solid electrolytes (ISEs), and organic–inorganic composite solid electrolytes (CSEs).

Solid-state electrolytes (SSEs) are the key components in solid-state batteries. It is important to obtain solid electrolyte materials with high ionic conductivity (≥10^−3^ S cm^−1^) and very low electronic conductivity (<10^−10^ S cm^−1^) [15], a wide electrochemical oxidation window, good chemical compatibility with the relevant electrodes, excellent thermal stability and mechanical properties [16], and that are easy to manufacture on a large scale and at a low cost. In the past few decades, three kinds of SSEs have been actively studied, including solid polymer electrolytes (SPEs), inorganic solid electrolytes (ISEs), and organic–inorganic composite solid electrolytes (CSEs). Great progress has been achieved in regard to synthesis and the ionic conductive mechanism. The properties of solid polymer electrolytes (SPEs), inorganic solid electrolytes (ISEs), and organic–inorganic composite solid electrolytes (CSEs) are shown in Figure 2.

### 2.1. Solid Polymer Electrolytes

**Figure 3:**
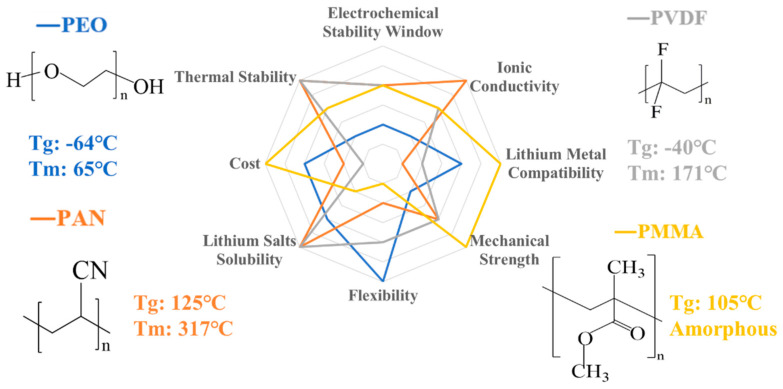
A comparison of the properties and structure of various polymer matrix materials.

Solid polymer electrolytes, such as poly (ethylene oxide) (PEO), were first reported by Wright in 1973 [17]. Nevertheless, the application of SPEs to lithium batteries was studied by Armand’s research group for the first time in 1983, inspiring intense research efforts in this field [18]. SPEs include dual-ion polymers and single-ion polymers. Dual-ion polymer electrolytes can be prepared by mixing the polymer and lithium salt, while single-ion polymer electrolytes involve lithium ion pairing with an anion on a segment of the modified polymer chain to form an ionic bond [19,20]. As a solid polymer electrolyte, functional groups in the polymer, such as -O-, -NH-, or -CN, are necessary to coordinate the lithium ions. These groups provide appropriate coordination sites to enable the dissociation of salts (i.e., solvating effect) and generate suitable sites adjacent to the mobile Li^+^ species for fast hopping [21]. The main polymer matrix materials used for polymer electrolytes include PEO [22], PAN [23], PVDF [24,25], and PMMA [26]. A comparison of their properties and structure is shown in Figure 3. Common lithium salts in solid-state polymer electrolytes are LiPF_6_, LiFSI, and LiTFSI, etc. The lone pair of electrons on the oxygen atom of PEO can coordinate with the lithium ions and the metal salt ions can be transported over a long distance with the breaking and formation of the “Li^+^-O” coordination bond and the continuous chain segment rearrangement. PEO is a semi-crystalline polymer, which has crystalline and amorphous states. The lithium ion transportion only occurs in the amorphous polymer region at room temperature. An increase in temperature results in a larger proportion of the amorphous region and a more intense “molecular chain segment motion”, which makes the lithium ions migrate faster and results in higher ionic conductivity being obtained. Other polymer matrix materials are like PEO and have the same lithium-ion migration mechanism.

**Figure 4:**
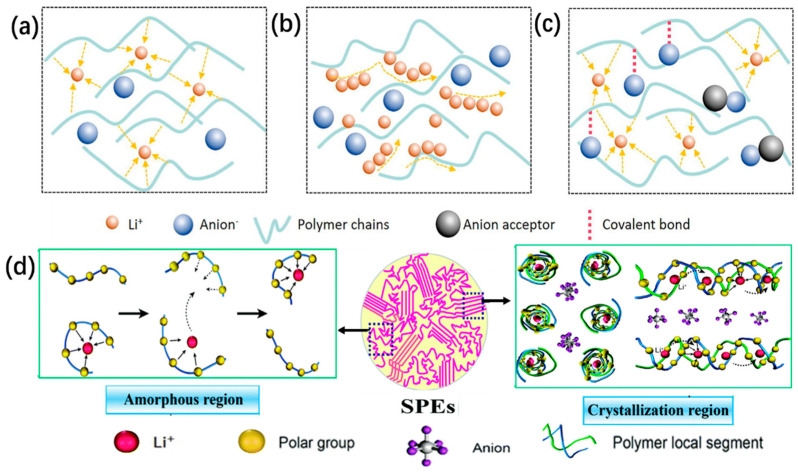
Schematic diagrams of various polymer electrolytes with the related ion migration mechanisms: (**a**) “salt-in-polymer”, (**b**) “polymer-in-salt”, and (**c**) “single-ion conducting” [31] electrolytes. Copyright 2021, Elsevier. (**d**) Scheme of Li-ion conduction model involving SPEs during the amorphous and crystalline phases [42]. Modified with permission from ref. [42], copyright 2016, Royal Society of Chemistry.

The transport mechanism of lithium ions in polymer electrolytes involves the lithium ions being conducted with the movement of the polymer chain segments. SPEs are mainly composed of a polymer matrix with a relatively small proportion of lithium salts (less than 50%), known as “salt-in-polymer” electrolytes (Figure 4a), which have low ionic conductivity [27,28,29,30]. Comparably, it was proposed that “polymer-in-salt” electrolytes have higher ionic conductivity, due to higher lithium salt content (over 50 wt.%) being employed in the polymer matrix (Figure 4b) [31]. Moreover, to prevent anions from migrating and to reduce the concentration polarization, single-ion (Figure 4c) conducting SPEs with a high ion transference number (t_Li+_) are designed in order to set anion traps or bind anions to the polymer matrix [9,20,32,33]. Meanwhile, the ion transfer rate of the polymer electrolyte is also influenced by amorphous and crystalline phases (Figure 4d). The migration of lithium ions is related to the free volume and transport properties of the polymer and the migration of macromolecular fragments during the solvation–dissolution process. Lithium ions will migrate from one coordination site to another or jump from one coordination site to another, along the direction of the polymer segment [34,35]. Specifically, lithium-ion transportation occurs primarily in the amorphous state, due to its lower glass transition temperature (Tg). Above the Tg, the chain segments begin to move, while the molecular chains do not. Thus, amorphous polymers in disordered environments allow local chain motion, which produces adjacent coordination sites, according to which ions can migrate [36,37]. In contrast, lithium ions in the crystallization section of the polymer electrolyte conduct Li^+^ through an ion jump, while the anions lie outside these channels and are separated from the cations by the interchain space [38,39]. For the migration of lithium ions in the amorphous and crystalline phases, there are differences and contradictions in different studies on the size of ionic conductivity in the crystal domain [40,41]. Most authors support the idea that a reduction in the Tg and an increase in the amorphous state of the polymer can improve the lithium-ion conductivity.

**Figure 5:**
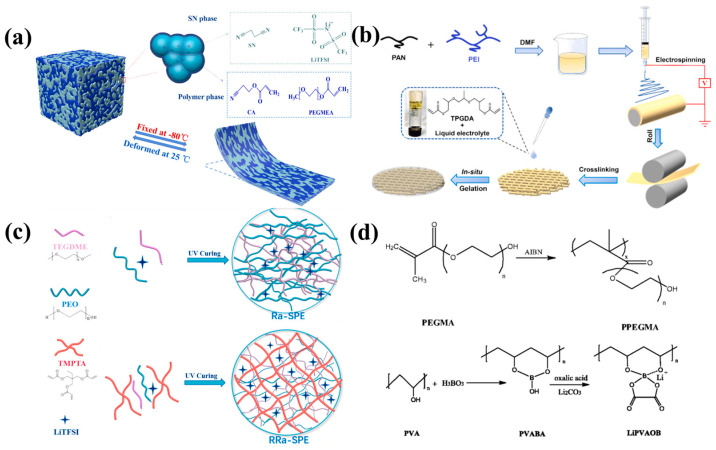
Preparation methods for different polymer electrolyte membranes: (**a**) CA-PEGMEA-SN solid polymer electrolyte prepared by in situ thermal-induced crosslinking polymerization [52], copyright 2024, Elsevier; (**b**) polymer electrolyte prepared by polymer spinning [50], copyright 2021, Elsevier; (**c**) the polymer electrolyte was prepared through the in situ UV-curing crosslinking method [53], copyright 2021, Elsevier; and (**d**) the single-ion polymer electrolyte was prepared by mixing PPEGMA/LIPVAOB [49], copyright 2018, Springer.

It is well-known that SPEs have superior processing capabilities and flexible properties. Compared to traditional organic liquid electrolytes, SPEs have the advantages of being light weight, low cost, non-flammable, offering good flexibility, superior mechanical and processing properties, and uniform lithium deposition [11,43,44]. Despite these advantages, the commercial application of SPEs is hindered due to its low ionic conductivity at room temperature. Several strategies have been adopted to improve the ionic conductivity of SPEs, such as crosslinking [45], blending [46], grafting [47], adding plasticizers [48], and increasing the temperature [49]. The addition of inorganic nano-ions or inorganic solid electrolytes to form composites is also a promising way to enhance the ionic conductivity of polymers, which will be mentioned in detail in Section 2.3. Simultaneously, the addition of ionic liquids [50] is also used to upgrade the ionic conductivity of solid polymers, by forming gel electrolytes [51]. Various polymer electrolytes have been synthesized, by adopting various ways to improve its ionic conductivity (Figure 5). Sun et al. [52] prepared polymer bi-phase SSEs, with a room-temperature ionic conductivity of 1.9 mS cm^−1^ (Figure 5a), through the in situ thermal-induced crosslinking polymerization of the elastomer monomer CA, the EO monomer PEGMEA, and the highly conductive SN phase. Wang et al. [50] prepared a PAN-PEI based electrolyte, with different mass ratios of PAN/PEI, using the electrospinning method to obtain a high level of ionic conductivity, up to 3.39 mS cm^−1^, at room temperature (Figure 5b). Fu et al. [53] reported on a SPE prepared via a facile UV-derived dual-reaction, which showed a high level of ionic conductivity of 4.36 × 10^−4^ S cm^−1^ at 30 °C (Figure 5c). Bozkurt et al. [49] synthesized a novel single-ion conducting polymer electrolyte based on lithium polyvinyl alcohol oxalate borate (Li(PVAOB)) and poly(polyethylene glycol methacrylate) (PPEGMA). The single-ion polymer electrolyte has a maximum ionic conductivity of 3 × 10^−4^ S/cm at 100 °C. Meanwhile, the insertion of more flexible skeletons into the solid polymer electrolyte can promote the movement of the chain, thereby increasing the transference number of the lithium ions. The ionic conductivity and the transference number of some polymers, as well as the test conditions and electrochemical properties, are listed in Table 1.

In summary, the matrix of solid polymer electrolytes has excellent elasticity, flexibility, elastic modulus, and interface contact properties, which can withstand the volume expansion and contraction of the electrode during battery charging and discharging. At the same time, SPEs can maintain good contact with the electrode to reduce the interface impedance, while ensuring the stability of the solid-state battery during the charge and discharge cycle. Polymer electrolytes are a solid electrolyte material with commercial feasibility and they have been used in a small range of new-energy vehicle power batteries [54]. However, low ion conductivity is still the main bottleneck in the development of SPEs. Obtaining high ionic conductivity and achieving a comprehensive level of performance are still the focus areas of future research on polymer electrolyte materials. Based on the relevant research progress, the future development of polymer electrolytes can be carried out in regard to the following aspects: (1) reduction of the molecular chain length and the crystallinity of the polymer chain segments and the improvement of mobility, while ensuring good mechanical strength; (2) the introduction of hyper delocalized groups into the molecular chain segment to upgrade the ionic conductivity at room temperature; (3) the insertion of a low Tg, flexible skeleton to promote the movement of the chain and, thus, increase the lithium ion transference number; (4) the addition of hydrophobic modules to ensure the mechanical strength and induce self-assembly to form more lithium-ion channels; (5) the addition of crosslinking active sites to allow crosslinking after self-assembly; (6) the introduction of a plasticizer to reduce the crystallinity and improve the conductivity without affecting the transfer of lithium ions; and (7) improvement of the safety of the battery interface, interface stability, and compatibility through in situ modification to form an artificial, solid electrolyte interface layer with rapid lithium-ion conduction to reduce the amount of stress between the electrolyte and the electrode.

**Table 1:** The ionic conductivity and the transference number of some polymers, as well as the test conditions.

Polymer Matrix	Li Salt	Temp. (°C)	Ionic Conductivity (S cm^−1^)	t_Li+_	Capacity mA h g^−1^ (Current Density, Cycle Index) (Test Temperature)	Refs.
CA/PEGMEA/SN	LiTFSI	25	1.9 × 10^−3^	0.56	171 (0.05C, 150) (25 °C)	[52]
TEGDME/PEO/TMPTA	LiTFSI	30	4.36 × 10^−4^	0.76	141.2 (0.5C 240) (30 °C)	[53]
PPEGMA	Li(PVAOB)	100	3 × 10^−4^	/	/	[49]
PEO	LiFSI	30	2.8 × 10^−4^	/	145.5 (0.5C, 750) (30 °C)	[55]
PTHF	LiClO_4_	60	2.3 × 10^−4^	0.36	142 (0.1C, 100) (60 °C)	[56]
PEC/PTMC	LiFSI	50	~10^−5^	0.6	150 (0.1C, 10) (50 °C)	[57]
PCL/PVDF	LiTFSI	60	~1.38 × 10^−4^	0.89	~112 (3C cm^−2^, 500) (60 °C)	[58]
PS-b-POEG_9_MA	LiClO_4_	25	10^−5^	/	~60 (8 mA g^−1^, 1) (20 °C)	[59]
PVDF-HFP	LiTFSI	70	7.24 × 10^−4^	0.57	150.6 (0.5C, 500) (70 °C)	[60]
PEO/PEG	LiTFSI	55	~10^−4^	/	127.7 (0.2C, 50) (55 °C)	[61]
PC	LiBNMB	22	2.5 × 10^−6^	/	--	[62]
PEO	P(SSPSILi-alt-MA)	25	3.08 × 10^−4^	0.97	~118 (0.1C, 350) (80 °C)	[63]
PEGMA/PEGDA	LiClO_4_	30	6.77 × 10^−5^	/	132 (0.1C, 40) (60 °C)	[64]
PEO/PEGDMA750	LiClO_4_	20	2.82 × 10^−5^	0.3	130.5 (0.1C, 150) (60 °C)	[65]
ePPO	LiTFSI	25	2.5 × 10^−4^	/	144 (0.2C, 300) (25 °C)	[66]
PI/PEO	LiTFSI	30	2.3 × 10^−4^	/	~125 (0.5C, 200) (60 °C)	[11]
PEO	LiDFOB	45	1 × 10^−5^	/	/	[67]
VDIM-TFSI/PVdF-HFP	LiTFSI	25	7 × 10^−4^	/	125.9 (0.1C, 100) (25 °C)	[68]
PEO-b-PA	LITFSI	25	3.7 × 10^−4^	0.57	136.8 (0.2C, 100) (25 °C)	[69]

### 2.2. Inorganic Solid Electrolytes

Inorganic solid electrolytes, including crystalline, partial crystalline (glass–ceramics), and amorphous glasses, exhibit the highest thermal stability and ionic conductivity among solid electrolytes. The activating energy of ISEs is less than 0.5 eV (<0.5 eV) and their ionic conductivity is higher than 10^−2^ S cm^−1^, being equivalent to that of liquid electrolytes at operating temperature. Based on their composition, ISEs can be divided into oxide, sulfide, and halide electrolytes. The ionic conductivity, advantages, and disadvantages of different types of inorganic solid electrolytes are listed in Table 2.

**Figure 6:**
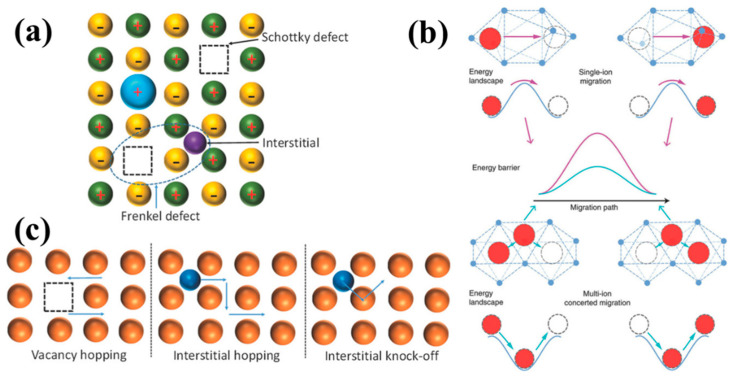
Mechanism of ion transfer in inorganic solid electrolytes [80]: (**a**) defect, (**b**) migration pathway, and (**c**) migration mechanism. Copyright 2018, Wiley.

The lithium-ion conduction mechanism of ISEs is realized through vacancy hopping of mobile ions (Figure 6). The vacancy mechanism relies on Schottky group defects (Figure 6a) [76], which provide many vacancies for ions to jump into the crystal. Vacancy ion diffusion requires mobile ions to occupy more equivalent (or approximately equivalent) positions than the number of mobile ions in the crystal frame structure, involving a simple vacancy gap mechanism and a gap substitution exchange mechanism (Figure 6b,c) [77,78,79]. In regard to the gap mechanism and the gap substitution exchange mechanism, gap ion diffusion occurs through the Frenkel defect, allowing lithium ions to migrate to nearby available locations.

#### 2.2.1. Solid Oxide Electrolytes

**Figure 7:**
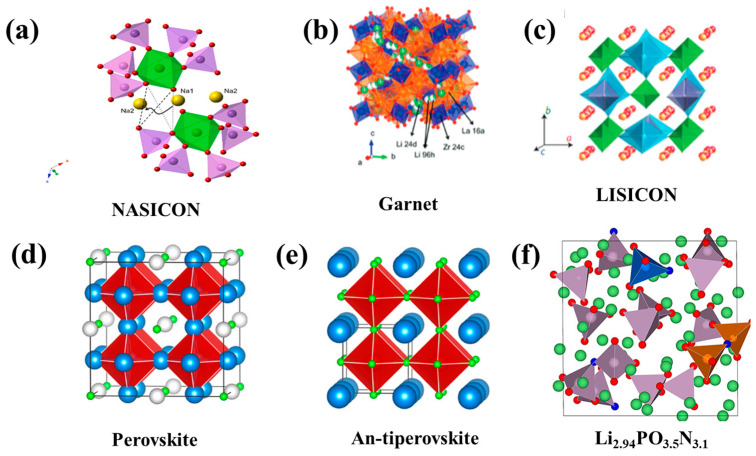
Typical structural families of oxide SEs: (**a**) NASICON [101]. copyright 2023, Elsevier; (**b**) garnet and (**c**) LISICON [80], copyright 2018, Wiley; (**d**) perovskite and (**e**) anti-perovskite [94], copyright 2023, Elsevier; (**f**) LiPON [102], copyright 2018, American Chemical Society.

Solid oxide electrolytes typically include NASICON-type [81], garnet-type [82], LISICON-type [70], perovskites [13], and anti-perovskites [83], and LiPON group [84] electrolytes, as shown in Figure 7. The NASICON-type electrolyte has a general formula, namely Li_1+x_MxTi_2−x_(PO_4_)_3_ (M = Al, Cr, Ga, Ge, Sc, In, Lu, Y, or La) [85] (Figure 7a). NASICON’s three-dimensional framework structure has high ionic conductivity, excellent structural stability, and a wide electrochemical stability window [79,86,87]. The solid NASICON electrolyte Li_1+x_Al_x_Ge_2−x_(PO_4_)_3_ (LAGP)/Li_1+x_Al_x_Ti_2−x_(PO_4_)_3_ (LATP), with a Li^+^ conductivity of 10^−3^ S cm^−1^, was prepared by doping Al on LiGe_2_(PO_4_)_3_ /LiTi_2_(PO_4_)_3_ [88]. Mohammadi et al. [89] prepared the Li_1.5_Al_0.4_Cr_0.1_Ge_1.5_(PO_4_)_3_ solid electrolyte, with a Li^+^ conductivity of 6.65 × 10^−3^ S cm^−1^.

Garnet-type SSEs exhibit high ionic conductivity (10^−4^–10^−3^ S cm^−1^), high stability in regard to anodes made of material like lithium metal, and high oxidation stability (up to 6 V vs. Li^+^/Li) (Figure 7b). For instance, Li_7_La_3_Zr_2_O_12_ (LLZO) exhibits a Li^+^ conductivity of 0.3 mS cm^−1^ at room temperature [90]. Considerable efforts have been devoted to enhancing the ionic conductivity and reducing the sintering temperature of LLZO through doping with other elements, such as Ta, Al, Ga, Nb, W, and Te, owing to the high stability and promising ionic conductivity of these elements [91]. Li et al. [92] reported that Li_7−x_La_3_Zr_2−x_Ta_x_O_12_ exhibited a high level of Li^+^ conductivity of 10^−3^ S cm^−1^ at room temperature. Qin et al. [93] prepared a highly textured Ga_2_O_3_-substituted Li_7_La_3_Zr_2_O_12_ compound, Li_6.55_Ga_0.15_La_3_Zr_2_O_12_, showing an ionic conductivity of 2.06 × 10^−3^ S cm^−1^ at room temperature. The LLZO-type SE has become one of the most promising inorganic solid electrolytes, due to its wide potential electrochemical window range, its good stability within different chemical and atmospheric environments, its low cost, the ease of availability of the starting materials, and its good thermal stability. The crystalline structures in lithium superionic conductor (LISICON) solid electrolytes are like γ-Li_3_PO_4_ (Figure 7c). The ionic conductivity of LISICON electrolytes in an oxide state is quite low at room temperature, about 10^−7^ S cm^−1^ [94]. Nevertheless, the ionic conductivity of LISICONs can be promoted to 10^−2^ S cm^−1^ at room temperature by replacing oxygen with sulfur, due to the introduction of lithium vacancies, such as Li_4_GeS_4_, Li_3_PS_4_, and Li_10_GeP_2_S_12_ [95].

Perovskite-type structured SEs have the same structure as CaTiO_3_, with a structural formula of ABO_3_ (A = Li, La; B = Ti) (Figure 7d). Li_3x_La_2/3−x_TiO_3_ (LLTO) is mainly studied due to its high ionic conductivity at ambient temperatures, its wide electrochemical windows, and its high oxidation stability [96]. The high grain boundary resistance of LLTO leads to its low total Li^+^ conductivity. In addition, LLTO will react with lithium metal to reduce Ti^4+^ to Ti^3+^, resulting in the decomposition of the electrolyte and short-circuiting of the battery. To improve the Li^+^ conductivity and cyclic performance of LLTO, element doping or the optimization of the Li content and the sintering temperature can be carried out to reduce the grain boundary impedance of LLTO and cause the reduction of Ti^4+^ [97,98].

In 2012, a new class of 3D-structured anti-perovskites (Figure 7e) was presented by Zhao et al. [99], namely “lithium-rich anti-perovskite [LiRAP]” solid-state electrolytes, with a formula of X^+3^B^2−^ A^−^ (e.g., Li_3_OCl). Within this structure, oxygen is at site B and any halogen [F, Cl, Br, I] or a mixture of halogens occupies site A. LiRAPs have reasonably high Li^+^ conductivity and high stability in regard to anodes made of materials like lithium metal. Unfortunately, the sensitivity of LiRAPs to water and the difficulty of synthesizing them in ambient atmospheres hinder their practical application.

Lithium phosphorus oxynitride (LiPON) has a general formula of Li_x_PO_y_N_z_ (Figure 7f). Compared to other solid electrolytes, LiPON is less sensitive to air and has high stability when in contact with lithium metal (up to 5.5 V). The literature-reported values on LiPON’s Li^+^ conductivity do not go beyond the order of 10^−5^ cm S^−1^ at room temperature [100]. Because the SSEs of LiPON have good stability in combination with lithium metal negative electrodes, the deposition rate can be controlled below 1 μm by sputtering and can be used as a protective layer for the Li metal negative electrodes.

#### 2.2.2. Sulfide Solid Electrolytes

**Figure 8:**
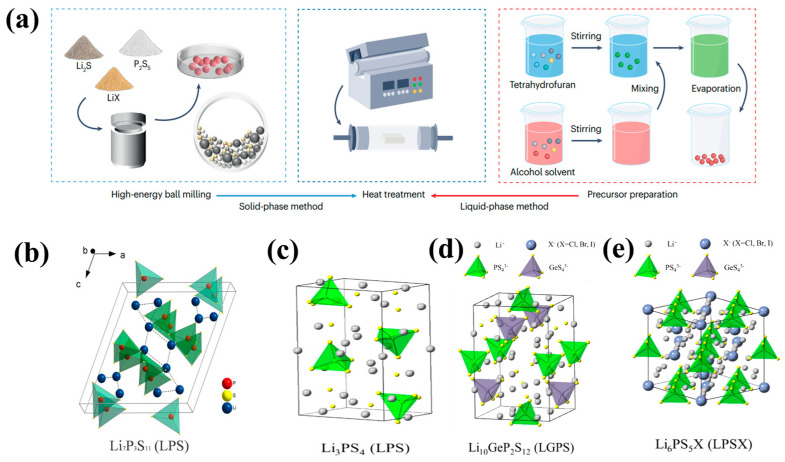
(**a**) Main preparation procedures [110], Copyright 2023, Nature Research. Crystal structures of sulfide SSEs: (**b**) Li_3_PS_4_ (LPS), (**c**) Li_7_P_3_S_11_, (**d**) Li_10_GeP_2_S_12_ (LGPS), and (**e**) Li_6_PS_5_X (LPSX) [111,112], copyright 2016, Elsevier, and copyright 2023, Springer Nature.

Sulfide is an amorphous system, possessing similar ionic conductivity to the electrolytic liquid phase. The sulfur atoms in sulfide electrolytes have lower electronegativity and larger ionic radii compared to oxygen atoms, resulting in weaker sulfur binding to lithium and faster lithium-ion transportion. Sulfide SEs can be prepared by solid-phase high-energy ball milling [103,104] or liquid-phase ball milling, and then heating quenching, forming an effective electrode–electrolyte interface [105,106] (Figure 8a). The ionic conductivity of sulfide SEs has exceeded 1 mS cm^−1^ and some can even reach more than 20 mS cm^−1^ at room temperature. In addition, sulfide electrolytes have low grain boundary resistance and good mechanical deformation ability, good contact with the electrode, and offer scalable cold-pressed fabrication. These advantages make sulfide SEs the most promising SE candidate of all the solid-state lithium batteries. However, sulfide SEs are sensitive to air and will decompose due to toxic gases in the air, which presents serious safety problems.

**Figure 9:**
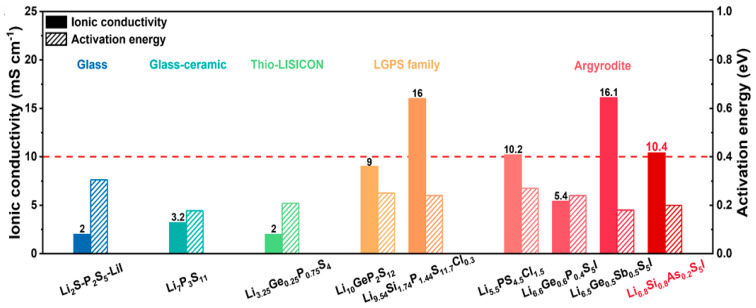
A comparison of ionic conductivity at 25 °C and activation energy of representative sulfide SEs reported in previous works [127], copyright 2023, Nature.

Sulfide solid electrolytes can be divided into pseudo-binary systems (Li_2_S-M_a_S_b_, M = P, Si, Sn, and Ge, etc.), pseudo-ternary systems (Li_2_S-P_2_S_5_-M_a_S_b_ and Li_2_S-P_2_S_5_-LiX, X = halogen, M = Ge, Sn, Si, Al, etc.), and pseudo-quaternary systems (e.g., Li_2_S + P_2_S_5_ + M_a_S_b_ + LiX). In addition, based on their structure and crystallinity, sulfide SEs can also be classified into three different kinds: glass, glass–ceramics, and crystalline. In 1981 [107], sulfide glass (Li_2_S-P_2_S_5_-LiI) was initially studied as a form of sulfide SEs. The glassy state of sulfide has low ionic conductivity, while the presence of crystals in sulfide electrolytes can lead to a high level of ionic conductivity. Hence, most high-ion-conductivity sulfide SEs reportedly belong to crystalline SEs, including thio-LISICONs (e.g., Li_4_GeS_4_ and Li_3_PS_4_), Li_10_GeP_2_S_12_-type SEs, and lithium argyrodites. The crystal structure of four typical sulfide SSEs, namely Li_7_P_3_S_11_, Li_3_PS_4_ (LPS), Li_10_GeP_2_S_12_ (LGPS), and Li_6_PS_5_X (LPSX), are shown in Figure 8b–e, and the ionic conductivity of the different types of sulfide electrolytes are listed in Figure 9. Due to the existence of grain boundary resistance, crystalline sulfide electrolytes can only exert high ionic conductivity in high-temperature or high-pressure conditions [108]. Sun et al. [109] reported on a novel amorphous SSE, 5Li_2_S-3SiS_2_ (mol %), with a high ionic conductivity of 1.2 mS cm^−1^.

Typical pseudo-binary SEs are the Li_2_S-M_x_S_y_ (M = P, Si, Sn, and Ge, etc.) system, such as Li_3_PS_4_, Li_2_S-SiS_2_. Li_3_PS_4_ is the most stable chemical in the Li_2_S-M_x_S_y_ system. The β-Li_3_PS_4_ phase exhibits the highest Li^+^ conductivity (Figure 8b). The zigzag arrangement of the PS_4_^3−^ tetrahedron can lead to more locations for Li^+^ movement, resulting in a high level of ionic conductivity during the β-Li_3_PS_4_ phase [113]. Yang et al. [114] proposed an Al and O dual-doped strategy for Li_3_PS_4_ to regulate the chemical/electrochemical stability of the anionic PS_4_^3−^ tetrahedra, to mitigate structural hydrolysis and the parasitic reactions at the SE/Li interface. The optimized Li_3.08_Al_0.04_P_0.96_S_3.92_O_0.08_ SE presents the highest Li^+^ conductivity of 3.27 mS cm^−1^, ~6.8 times higher than the pristine Li_3_PS_4_, and the Li_3.08_Al_0.04_P_0.96_S_3.92_O_0.08_ SE effectively inhibits structural hydrolysis for ~25 min, @ 25% humidity, at room temperature. Yamane et al. [115] developed a new and highly conductive Li_7_P_3_S_11_, with a conductivity of 3.2 × 10^−3^ S cm^−1^ at room temperature. Li_7_P_3_S_11_ is made up of a P_2_S_7_ ditetrahedra and a PS_4_ tetrahedra (Figure 8c), having an anionic subshell, with a framework like bcc. The rapid diffusion of lithium ions is facilitated by the direct jump between adjacent tetrahedral sites [116]. In addition, based on the hard and soft acids and bases (HSAB) theory, the air stability of sulfide SEs can be improved by completely replacing the hard acid (P) in the sulfide with soft acids, such As, Sb, and Sn [117]. Kaib et al. [118] reported that the ion conductivity of Li_4_SnS_4_ was 7 × 10^−5^ S cm^−1^ at 20 °C and 3 × 10^−3^ S cm^−1^ at 100 °C and the air stability of the electrolyte was improved by substituting P with Sn. To improve the ionic conductivity, Kanno et al. [119] synthesized Li_2_SiS_3_ with tetragonal crystal symmetry, achieving a high level of Li^+^ conductivity of 2.4 mS cm^−1^ at room temperature.

Pseudo-ternary systems (Li_2_S-P_2_S_5_-M_a_S_b_ and Li_2_S-P_2_S_5_-LiX, X = halogen, M = Ge, Sn, Si, Al, etc.), such as Li_2_S-GeS_2_-P_2_S_5_, belong to thio-LISICON group. Thio-LISICONs also have a β-Li_3_PO_4_ framework structure. Because the attraction between S^2−^ and Li^+^ in LISICONs is weaker than that between O^2-^ and Li^+^, thio-LISICONs exhibit higher ionic conductivity (10^−3^ S cm^−1^ at 25 °C) than oxide LISICONs [120,121]. In addition, the increase in ionic conductivity of thio-LISICONs is due to vacancy doping and gap doping. When such doping produces a Li^+^ vacancy or Li^+^ interstitials, a significant enhancement in the ionic conductivity can be observed. Kanno et al. [122] obtained Li_3.25_Ge_0.25_P_0.75_S_4_, with a Li^+^ conductivity of 2.2 mS cm^−1^ at 25 °C, in 2001. Li_10_GeP_2_S_12_ has a three-dimensional diffusion path along the c-axis and the a–b plane, due to its three-dimensional crystal structure (Figure 8d), illustrating a good level of ionic conductivity, close to that of organic state electrolytes at room temperature, and an electrochemical window of 5 V vs. Li/Li^+^ [123]. However, the low reserves and the high price of germanium limit the application of LGPS. The cost can be greatly reduced by substituting Ge with Sn, with the ionic conductivity kept at 4.79 × 10^−3^ S cm^−1^ at room temperature [124].

Lithium argyrodites (Li_6_PS_5_X, X = I, Br, Cl) (Figure 8e) have been widely studied because of their low cost, high Li^+^ conductivity, and good dynamic stability in regard to Li metal anodes. Li_6_PS_5_X (X = Br and Cl) has a high level of ionic conductivity (4.96 mS cm^−1^ for Li_6_PS_5_Cl and 3.9 mS cm^−1^ for Li_6_PS_5_Br) [125,126], due to the high proportion of anion site disturbances in the S^2−^ and X^−^ positions in Li_6_PS_5_X (X = Cl and Br). The ionic conductivity of Li_6_PS_5_I is very low (4.6 × 10^−4^ mS cm^−1^), because there are no anion site disturbances in Li_6_PS_5_I.

Pseudo-quaternary systems (e.g., Li_2_S + P_2_S_5_ + M_x_S_y_ + LiX) have higher ionic conductivity than pseudo-ternary system electrolytes, as they incorporate multiple elements. A series of new sulfide SSEs with enhanced ionic conductivity and interfacial stability have been gradually designed through structural and component tuning. Kanno et al. [128] reported that Li_9.54_Si_1.74_P_1.44_S_11.7_Cl_0.3_ has an exceptionally high level of conductivity (25 mS cm^−1^) and stability (∼0 V versus Li metal for Li_9.6_P_3_S_12_). Liang et al. [129] prepared Li_10_Ge(P_0.925_Sb_0.075_)_2_S_12_, which has a high level of ionic conductivity of 17.3 mS cm^−1^ at ambient temperature, by replacing part of the P element with Sb. Due to aliovalent doping of the Li^+^ site with Ca^2+^, vacancies were created to improve Li ion diffusion, resulting in the high ionic conductivity of the sulfide SEs. Li et al. [130] designed a highly ion-conductive solid electrolyte by increasing the compositional complexity of a known lithium superionic conductor to eliminate ion migration barriers, while maintaining the structural framework for superionic conduction. Sun et al. [131] reported a novel cube-shaped microstructure within the Li_5.3_PS_4.3_ClBr_0.7_ argyrodite electrolyte, exhibiting a higher level of ionic conductivity of 14.1 ± 0.1 mS cm^−1^. Adeli et al. [132] synthesized “super Cl-rich” material, with an overall composition of Li_5.35_Ca_0.1_PS_4.5_Cl_1.55_, exhibiting superionic room-temperature conductivity of 10.2 mS cm^–1^ in the cold-pressed state. Kanno et al. [130] designed a high-entropy Li_9.54_[Si_0.6_Ge_0.4_]_1.74_P_1.44_S_11.1_Br_0.3_O_0.6_ electrolyte, with ionic conductivity of up to 32 mS cm^−1^ at room temperature. The compositional complexity of the LGPS-type LSiPSCl superionic conductor was improved and the structural framework for superionic conduction was kept. A pathway for three-dimensional superionic lithium-ion conductivity was built using two anions to improve the ion conductivity of SSEs [133]. Although sulfide solid electrolytes have a high level of ionic conductivity, the instability caused by the side reaction with the lithium anode still needs attention. First, the chemical potential of sulfide solid electrolytes does not match the chemical potential of lithium anodes, and the two will directly and spontaneously react when they come into contact, consume active substances, generate a harmful SEI layer at the interface, and hinder the transport of ions at the interface. Second, the harmful products generated by the oxidation–reduction reaction of the sulfide solid electrolyte itself under the influence of the electric field are enriched at the interface, which then destroy the interface microstructure, resulting in an increase in the interface resistance.

#### 2.2.3. Halide Solid Electrolytes (HSEs)

**Figure 10:**
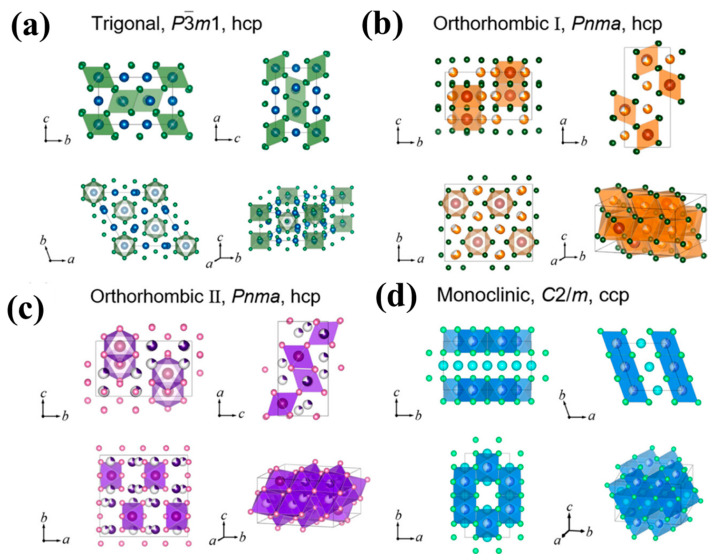
Crystal structures with a single unit cell (outlined) and the corresponding Li+ sublattice for: (**a**) a trigonal structure, (**b**) an orthogonal structure I, (**c**) an orthogonal structure II, and (**d**) a monoclinic structure [139]; copyright 2023, American Chemical Society.

The general chemical formula of halide solid electrolytes is Li_a_-M-X_b_ (X = Br Cl F, M = metal), being similar to the product obtained by introducing the high-valence transition metal element M cation into the lithium halide, LiX (X = Br Cl F) [134]. Li_a_-M-X_b_ compounds are promising materials because halides can achieve stable cycling in high voltage windows. Fluoride electrolytes with high ionic conductivity are rarely reported. Compared with bromide compounds, chloride compounds have attracted much attention due to their good deformability, excellent ionic conductivity, and high voltage stability. In regard to halides, their ionic radii are comparable to sulfide ions and are larger than oxygen ions. Ionic cooperation in metal halide electrolytes is weak, leading to a mobile state of the Li^+^ in most anionic lattice structures that is needed in order to obtain high Li^+^ conductivity. There are three common halide electrolytes: Li_a_-M-Cl_6_, Li_a_-M-Cl_4_, and Li_a_-M-Cl_8_. The ionic conductivity of the first two classes can reach 10^−3^ S cm^−1^, while Li_a_-M-Cl_8_ electrolytes have lower ionic conductivity and instability at room temperature (such as Li_6_FeCl_8_ [135], LiMOCl_4_ (M = Nb, Ta) [136,137,138]). According to the bonding of Li_a_-M-X_6_ metal halide electrolytes, the common crystal structure includes a trigonal crystal system (hcp-T) involving the P3m1 space group, an orthorhombic crystal system (hcp-O) involving the pnma space group, and a cubic crystal system (ccp) involving the C2/m space group, as shown in Figure 10a–d. Halide electrolytes can undergo phase transition at various temperatures, which affects its conductivity, and are easily hydrolyzed in air, making them costly to synthesize. In addition, the transition metals in halide electrolytes have a negative impact on the compatibility of lithium anodes, due to their reaction with lithium metals. Numerous strategies have been developed to improve the conductivity of Li ions in HSEs. Replacing or introducing cationic dopants with a lower or higher valence number is one of the most effective strategies to improve the conductivity of lithium ions. Introducing cation dopants with lower valence numbers can cause anion vacancies or cation interstitials, while doping with higher-valence cations can cause anion interstitials or cation vacancies.

**Figure 11:**
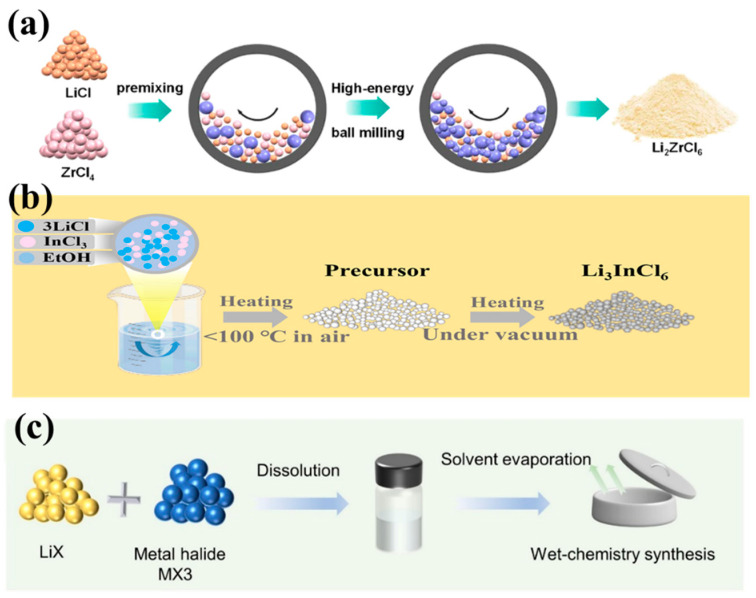
Synthesis methods for halide SEs: (**a**) high-energy ball milling [151], copyright 2024, American Chemical Society; (**b**) comelting [152], copyright 2022, American Chemical Society; (**c**) wet chemistry synthesis [153], copyright 2022, Science.

There are currently three proposed methods for the synthesis of halide electrolytes (Figure 11), including a mechanochemical method [140], melting [141] and wet chemical synthesis [142]. Li_3_InCl_6_ has received particular attention as a halide SE. Li et al. [143] achieved reversible conversion between Li_3_InCl_6_ and Li_3_InCl_6_·2H_2_O, which can maintain its structure and ionic conductivity when coming into contact with moist air and that leads to the high stability of Li_3_InCl_6_ in an ambient atmosphere. Lutz et al. [144] synthesized Li_3_InCl_6_, with a low ionic conductivity of ∼3.2 × 10^−5^ S cm^−1^ at 60 °C, by melting LiCl and InCl_3_. Sun et al. [145] prepared highly crystalline Li_3_InCl_6_, with an ionic conductivity of 1.49 × 10^−3^ S cm^−1^ at room temperature, using a ball-milling and post-annealing process. Luo et al. [146] found that mild heat treatment (100 °C) can apparently enhance the ionic conductivity of ball-milled electrolytes by 2–3 times, primarily attributed to the network-like micromorphology of the nanoparticles, which is beneficial for Li^+^ migration. The Li^+^ ionic conductivity of Li_2_ZrCl_6_ has a value of 4.46 × 10^−4^ S cm^−1^ at room temperature, when being gently heated. Li et al. [143] successfully prepared Li_3_InCl_6_, with a conductivity of 2.04 × 10^−3^ S cm^−1^ at 25 °C, in an aqueous solution. Sun et al. [147] achieved for the amorphous Li_3_ZrCl_4_O_1.5_, an ionic conductivity of (1.35 ± 0.07) × 10^–3^ S cm^–1^ at 25 °C. Yao et al. [148] prepared the Li_0.388_Ta_0.238_La_0.475_Cl_3_ electrolyte, with a conductivity of 3.02 mS cm^−1^ at 30 °C, and which had a low activation energy of 0.197 eV. Sun et al. [149] reported a new class of zeolite-like halide frameworks, which achieved an ionic conductivity of over 10^–4^ S cm^–1^ at 30 °C, with LiCl as the adsorbent. Sun et al. [150] reported a series of fluorinated lithium tantalum oxychlorides (LTOC-F) as amorphous SEs, which had a high ionic conductivity of 2.3 mS cm^–1^ at 25 °C. Wang et al. [139] found that ionic conductivity can be improved through the Hf substitution of Li_3_InCl_6_ (Li_3−x_In_1−x_Hf_x_Cl_6_, 0 ≤ x ≤ 0.7) SEs without affecting the electrochemical stability, even at a low concentration (0.1 ≤ x ≤ 0.5) of Hf. Among them, Li_2.7_In_0.7_Hf_0.3_Cl_6_ exhibited a high ionic conductivity of 1.28 mS cm^−1^ and a wide electrochemical stability window of 2.68–4.22 V. The ionic conductivity of halide solid electrolytes is listed in Table 3.

**Figure 12:**
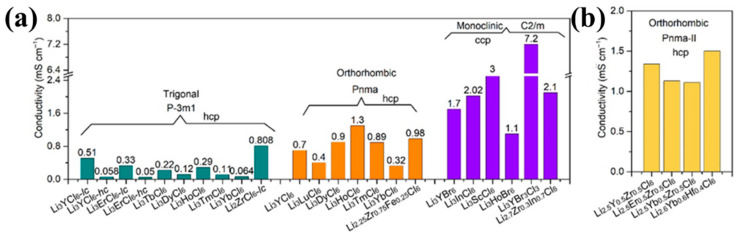
Ionic conductivity of halide SSEs: (**a**,**b**) room-temperature ionic conductivity of ternary halide electrolytes and quaternary halide electrolytes [153]; copyright 2022, Science.

To better understand the structure–activity relationship, the ionic conductivity of different halides is summed up according to the difference between the anionic sublattice and the spatial groups (as shown in Figure 12). Monoclinic halide SSEs with a ccp anion sublattice show much higher ionic conductivity than trigonal and orthorhombic halide SSEs with a hcp framework. The general trend of ionic conductivity is σ_monoclinic_ > σ_orthorhombic_ > σ_trigonal_. In summary, the particle size, micromorphology, and mechanical properties of halide electrolytes have a huge impact on the performance of SSEs. Therefore, it is of great significance to improve the ion conductivity and air stability of such SSEs by exploring the synthesis pathway and rational structural design for the practical application of halide electrolytes. In addition, high-entropy solid electrolytes have been the subject of some research. Luo et al. [161] reported a high-entropy Li_2.75_Y_0.16_Er_0.16_Yb_0.16_In_0.25_Zr_0.25_Cl_6_ that boosted the cycle stability of an all-solid-state battery, with an improvement of 250% over 500 cycles. Zeng et al. [162] demonstrated the ability of high-entropy metal cation mixes to improve the ionic conductivity of a compound, which leads to less reliance on specific chemistries and enhanced synthesizability. Xu et al. [163] presented a high-entropy electrolyte composed of lithium triflate (LiOTf) and trimethyl phosphate (TMP) co-added to magnesium bis(trifluoromethane sulfonyl)imide (Mg(TFSI)_2_/1,2-dimethoxyethane (DME) that significantly improved the electrochemical performance of Mg-metal anodes. Sun et al. [164] reported a series of UCl3-type SSEs with high room-temperature ionic conductivities of over 10^−3^ S cm^−1^ and good compatibility with high-voltage oxide cathodes. Brezesinski et al. [165] reported on medium- and high-entropy polyanionic lithium superionic conductors that crystallized the *F*–43*m* space group and adopted the so-called argyrodite structure.

### 2.3. Organic–Inorganic Composite Solid Electrolytes (Organic–Inorganic CSEs)

Organic–inorganic composite solid electrolytes (referred to as CSEs) are composed of a polymeric matrix, lithium salt, and an inorganic filler. The polymer matrix endows the composite solid electrolyte with good toughness, which reduces the interface impedance between the electrolyte and the electrode material. The inorganic filler improves the mechanical strength of the composite solid electrolyte by reducing the crystallinity of the polymer matrix, resulting in an increase in the lithium-ion migration rate by providing a fast channel for the lithium-ion migration. Composite solid electrolytes have a high level of ionic conductivity and a wide electrochemical oxidation window. Composite solid electrolytes overcome the problem of low conductivity experienced by polymer solid electrolytes and the problem related to poor electrolyte/electrode interface contact caused by the brittle hardness of inorganic solid electrolytes is alleviated. In response to the demand for electrolytes with a variety of comprehensive performance-related capabilities, organic–inorganic CSEs can be used in solid-state batteries and have certain advantages in order to meet the practical application requirements related to electrolyte materials.

**Figure 13:**
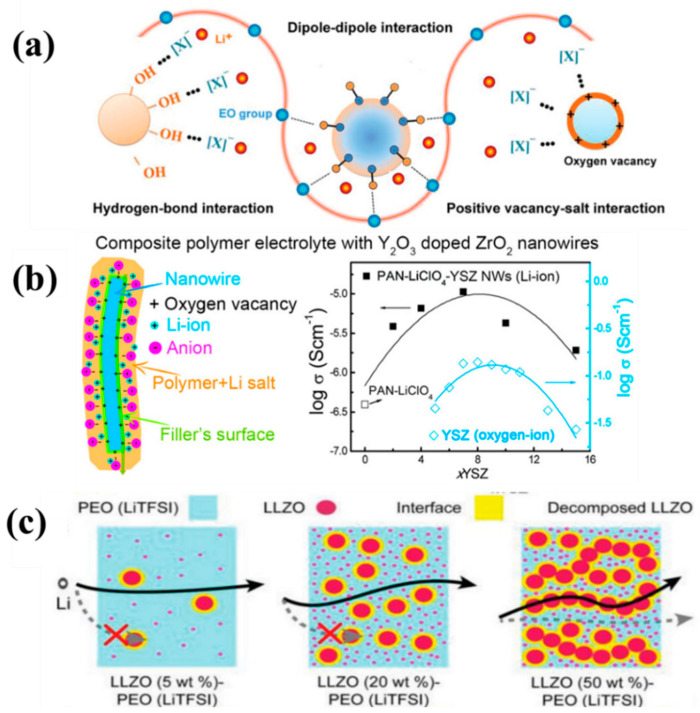
Ion transport in organic–inorganic composite electrolytes: (**a**) the Lewis acid-base interaction between different components in CSEs [170], copyright 2019, Wiley; (**b**) structure and ionic conductivity of PAN-LiClO_4_@Y_2_O_3_-doped ZrO_2_ CSEs [172], copyright 2016, American Chemical Society; (**c**) schematic diagram of Li^+^ ion diffusion routes in PEO-LiTFSI with different contents of LLZO fillers [173], copyright 2018, American Chemical Society.

Inorganic fillers can be divided into inert fillers and non-inert fillers (that is mainly inorganic electrolytes). Inert fillers, such as oxides (such as Al_2_O_3_ [166], SiO_2_ [167], TiO_2_ [168], and ZrO_2_ [169]), metal–organic frameworks, and clay minerals (such as montmorillonite, halloysite, etc.), can improve the ionic conductivity of composite electrolytes, mainly due to the fact that the inert filler can reduce the glass transition temperature (Tg) and the crystal composition of the polymer (such as PEO) by increasing the amorphous form of the polymerization (that is, the amorphous structure) [170]. The packing surface of inert fillers provides some paths for ion transfer that distorts the highly ordered polymer chain segments and increases the content of the amorphous phase. The Lewis acid-base interaction between the polymer chain and the inert fillers can inhibit the crystallization of the polymer chain and enhance the kinetics segment movement. As a result, the concentration of mobile lithium ions and ionic mobility are enhanced (Figure 13a) [167]. The interaction of hydrogen bonds between the filler and the polymer can break the regular arrangement of the conductive segments of the polymer matrix, which increases the amorphous region of the polymer, leading to an increase in the ionic conductivity. Therefore, the Lewis acid-base interaction between the filler and the polymer matrix allows the formation of an interface layer between the filler and the polymer to form a fast ion transport pathway. Nanoparticles and nanoporous materials (e.g., mesoporous silica pores, Al_2_O_3_ and metal–organic skeleton nanowires or layered materials) are helpful for Lewis acid-base interactions and the production of more interface layers, which leads to a greater increase in ionic conductivity (Figure 13b). Pal et al. [171] explored the impact of TiO_2_ nanoparticles on the characteristics of the PMMA-LiClO_4_-1wt%TiO_2_ composite solid electrolyte, obtaining an iconic conductivity of 3 × 10^−4^ S cm^−1^ at room temperature.

Non-inert fillers (that is inorganic electrolyte fillers) have a high level of ionic conductivity, such as LLZO [174,175] and sulfide [122]. Therefore, the ionic conductivity of composite electrolyte materials prepared by adding inorganic electrolyte fillers will be greatly improved, even the ionic conductivity of micron LLZO particles will largely be improved (Figure 13c). It is important to ensure that the surface is fresh when using LLZO particles, because LiOH and Li_2_CO_3_ are easily formed when exposed to air [176]. For composite electrolytes composed of an inorganic electrolyte filler and a polymer electrolyte, the inorganic electrolyte filler helps to form the interface layer, which creates an ion transport pathway between the polymer and the filler through the Lewis acid-base interaction, as well as helping to promote the rapid ion transport of lithium ions due to the high Li^+^ conductivity of the inorganic electrolyte filler itself. Although many experiments and simulations have been dedicated to revealing the ion transport behavior in regard to composite electrolytes, the mechanism of Li^+^ conduction is poorly understood because little is known about the complex interactions between the internal interface layers and various components, including inorganic filler polymers, such as lithium salts and plasticizer.

It has been reported that the particle size, concentration, morphology, orientation, surface modification, and small-molecule additives of inorganic fillers have a significant impact on the ionic conductivity of hybrid electrolytes, as depicted in Table 4. Fillers with large specific surface areas and small sizes, such as nanoparticles, nanofibers, or nanowebers, can facilitate Lewis acid-base interactions, which create a more continuous osmotic pathway for lithium-ion movement, provide longer dimensions, results in less aggregation variation, and improve the ionic conductivity, resulting in a higher lithium ion transference number for the composite polymer electrolyte [177]. The concentration of inorganic fillers also has a great impact on the ionic conductivity. When the concentration of the filler is very low, the ions will preferentially be transported during the polymer phase. When the concentration of the filler reaches a predetermined threshold, the lithium-ion conductivity increases in regard to the quantity of the filler. Conversely, when the concentration of the inorganic filler exceeds the penetration threshold, the lithium-ion conductivity decreases. Therefore, the incorporation of a suitable proportion of inorganic fillers is helpful to obtain a composite electrolyte with high ionic conductivity. Furthermore, it has been demonstrated in some studies that directionally arranged nanowires and three-dimensional (3D) integrated skeleton structures can prevent the accumulation of inorganic fillers, thereby providing a continuous lithium-ion transport channel to preserve the high level of ionic conductivity [178]. To enhance ion mobility and conductivity in composite electrolytes, some additives in the form of plasticizers were introduced, such as SN and TEGDME.

**Figure 14:**
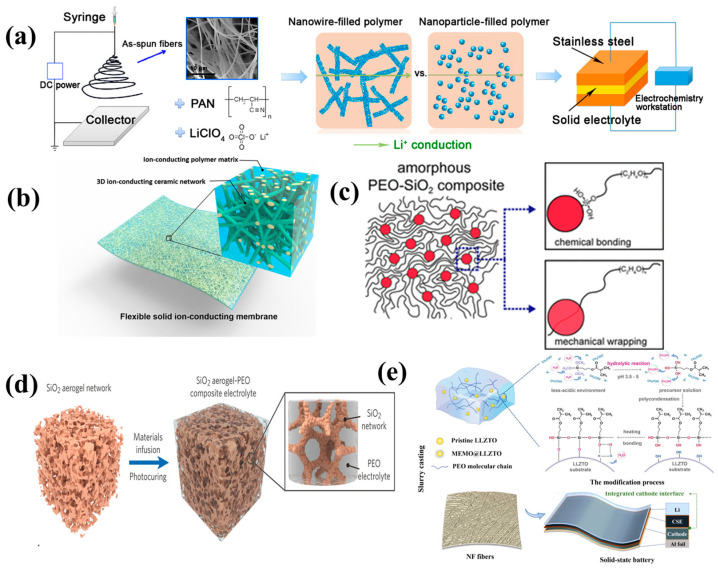
Schematics of CSEs including fillers with different morphologies. (**a**) An illustration of the synthesis of ceramic nanowire-filled polymer-based composite electrolytes [181]; copyright 2015, American Chemical Society. (**b**) An illustration of the procedure to synthesize flexible solid-state garnet LLZO nanofiber-reinforced polymer composite electrolytes [180]; copyright 2016, PNAS. (**c**) The CSEs of PEO/MUSiO_2_ NPs [179]; copyright 2016 American Chemical Society. (**d**) An illustration of the synthetic procedures involving SiO_2_-aerogel-reinforced CSEs [188]; copyright 2018, Wiley. (**e**) An illustration of an all-solid-state lithium battery with an MEMO@LLZTO-PEO electrolyte [189]; copyright 2024, Elsevier.

To construct a composite solid electrolyte with high ionic conductivity and excellent wetting ability, a polymer solution was directly injected into an inorganic electrolyte with a 3D continuous network structure to form an inorganic electrolyte penetration network, and then the solvent was evaporated [177]. Fast Li-ion transportation was achieved through the continuous network structure framework phase of the inorganic electrolyte. Additionally, by electrospinning or using a variety of templates, such as cellulose textiles, polyurethane foam, or 3D-printed templates, and then sintering, the resulting framework phase can facilitate the rapid transportation of ions. Although 3D networks created by different template methods are less productive, and electrospinning often requires harmful solvents, the strategy is useful for improving ion conductivity in composite electrolytes. For example, Cui et al. [181] synthesized polymer-based composite electrolytes involving ceramic-filled nanowires, exhibiting a very high ionic conductivity of 2.4 × 10^−4^ S cm^−1^ at room temperature (Figure 14a). Hu et al. [180] devised a 3D lithium-ion conducting ceramic network based on a garnet-type Li_6.4_La_3_Zr_2_Al_0.2_O_12_ (LLZO) lithium-ion conductor to provide continuous Li^+^ transfer channels in a polyethylene oxide (PEO)-based composite, exhibiting an ionic conductivity of 2.5 × 10^−4^ S cm^−1^ at an ambient temperature (Figure 14b). Lin et al. [179] reported that a composite electrolyte involving PEO and MUSiO_2_ achieved a high level of ionic conductivity of 1.2 × 10^−3^ S cm^−1^ at 60 °C (Figure 14c). Cui et al. [188] successfully designed and fabricated ultra-strong, reinforced composite polymer electrolytes by introducing a stiff mesoporous SiO_2_ aerogel as the backbone for the polymer-based electrolyte (Figure 14d). Kim et al. [183] synthesized a composite electrolyte membrane containing 7 wt% of LLZTO and 60 wt% of BMIMOTf, showing an outstanding Li^+^ conductivity of 2 × 10^−3^ S cm^−1^ at room temperature. According to Wu et al. [185], the in situ copolymerization of poly(ethylene glycol methacrylate)-Li_1.5_Al_0.5_Ge_1.5_(PO_4_)_3_-lithium (PEGMA-LAGP-Li) is a novel approach to making CSEs. The proposed hierarchy offers a promising synergy in terms of flexibility–rigidity (Young’s modulus 3 GPa) and high ionic conductivity (2.37 × 10^−4^ S cm^−1^). Song et al. [189] designed a Li_6.4_La_3_Zr_1.4_Ta_0.6_O_12_ (LLZTO) filler coated with a 3-methacryloxypropyltrimethoxysilane (MEMO) Janus layer for a poly(ethylene oxide) oxide (PEO) electrolyte, denoted as MEMO@LLZTO-PEO, Figure 14e, which exhibited an ionic conductivity of 2.16 × 10^−4^ S cm^−1^ at 30 °C.

In summary, the introduction of inorganic nanofillers can provide pathways for Li^+^ transfer on their surface, distort highly ordered polymer chain segments, and increase the content during the amorphous phase, providing the possibility of increasing the electrical conductivity of SSEs. Meanwhile, the addition of inorganic nanofillers can improve the mechanical properties of the electrode and the interface contact with the electrode, contributing to an increase in the amorphous phase of the polyelectrolyte. As a result, the composite solid electrolyte combines the advantages of the polymer and the inorganic electrolyte, and complements their drawbacks, making the composite solid electrolyte an essential component in the future preparation of solid-state batteries.

## 3. Challenges Concerning Solid-State Batteries

It is imperative to employ a combination of solid electrolytes, lithium metal anodes, and high-voltage cathode active materials (CAMs) to fabricate solid-state batteries with high-energy density. Nevertheless, electrode materials have a strong chemical reaction with electrolytes. Despite the advancements that have been made in the attainment of high ionic conductivity of solid electrolyte materials, the limitation consisting of high impedance at the interface between the electrolyte and the electrode, the substantial resistance and serious interface side reactions resulting from inadequate interface contact, and the lithium dendrite issue, are still the primary factors contributing to the suboptimal performance of solid-state batteries [177]. The current research on the solid-state battery interface focuses on the reduction of solid-state electrolyte grain boundary impedance and the elimination of the grain boundary, the improvement of the interface compatibility between lithium metal and solid electrolytes (side-reaction dendrite lithium empty layer), as well as the problems concerning the contact between positive electrodes and the solid electrolyte interface volume effect, the space-charge layer effect, and element diffusion, etc. The technical difficulties with solid batteries mainly relate to the high interface impedance between the solid electrolytes and the electrode, and the poor interface compatibility. During the charge and discharge process, the material’s volume expands and contracts, making it easy to separate the interface, which directly affects the low-temperature performance, fast-charging performance, energy density, and power density of the battery.

### 3.1. High Interfacial Resistance

**Figure 15:**
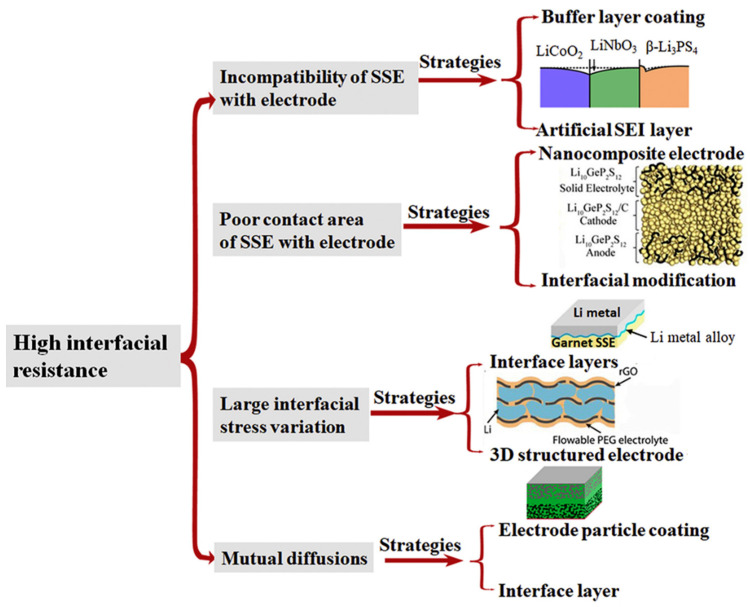
Strategies for reducing significant solid–solid interfacial impedances [202]; copyright 2019, Cell Press.

The high level of interface impedance hinders the rapid development of solid-state batteries. Because the interface between the electrode and the electrolyte is a solid-state contact point, the solid phase is not able to be wettable. The formation of higher interface resistance, which is not conducive to Li^+^ transmission, will ultimately reduce the battery’s power output capacity and the efficiency of the charge and discharge process. Furthermore, the high interface impedance is caused by electrochemical instability, space-charge effect, mutual diffusion, and other factors, due to the potential difference between the positive and negative electrodes and the electrolyte, which results in the transfer of lithium ions from the electrolyte to the other side. The positive and negative electrodes and the local lithium ion in the electrolyte lack a space-charge layer, resulting in a space-charge effect, which limits the battery’s rate performance [190]. The space-charge layer is a region subject to charge carrier concentration variations of two-phase interfaces, rather than distinct point-like charges, a natural bridge between the SSEs and the electrode, and it is often used to explain ionic conductivity in heterogeneous systems [191,192]. In particular, the space-charge layer in the sulfide solid electrolyte enlarges the impedance at its interface because, in a solid-state battery, the oxide cathode material used is a mixed conductor with high ionic conductivity, but the sulfide electrolyte is a single lithium-ion conductor. When the oxide cathode material comes into contact with the sulfide solid electrolyte, the large potential difference at the interface makes it easy for lithium ions to transfer from the sulfide electrolyte to the oxide electrode side. The effect of electron conduction can be mitigated and lithium ions continue to move to the cathode side, causing the space layer to expand further and ultimately resulting in high interface resistance. The side reaction at the interface between the lithium metal anode and the electrolyte will also enlarge the interface resistance, leading to the attenuation of the electrochemical performance of the battery. Furthermore, the interdiffusion layer of the elements formed at the electrolyte–electrode interface will affect the interface stability and increase the interface impedance, such as due to a phase shift or the formation of a resistance layer. Due to the rigidity of oxide-based solid electrolytes, the battery manufacturing process is often dependent on additional heating steps to strengthen the bonds between the electrode and the electrolytes. Thus, there is a mutual diffusion area in terms of the elements at the solid–solid interface and, inevitably, a large interface resistance is formed at the same time. Simultaneously, numerous internal micro-cracks will arise during the charge and discharge process, due to the continual expansion and shrinkage of the electrode material as a result of the Li^+^ being removed from or inserted onto the positive and negative electrode materials. The micro-cracks will lead to a reduction in the life span of the battery and an increase in the impedance between the positive and negative electrodes of the solid electrolyte. There have been several approaches proposed to solve the abovementioned problems, as shown in Figure 15. First, various thin films that form a buffer layer, such as LiNbO_3_ [193], LiPO_3_ [194], and Al_2_O_3_ [195], on the electrode particles have been proposed as an effective approach to reducing the incompatibilities. Introducing an artificial SEI layer [196] to promote electronic insolation and ionic conduction is an effective approach to reducing interface instability. Second, introducing an interface modification layer and the nanocomposites of electrodes (such as ball milling [197], atomic deposition [198], pulsed-laser deposition (PLD) [199] technologies to prepare the nanoparticles) van help to address the poor contact area between solid-state electrolytes and the electrode. Third, the use of composite electrodes with flexible, amorphous, gel-state interfaces helps in the mitigation of the large interfacial stress variations resulting from the large volume changes to the electrodes [200]. Fourth, the electrode interface being coated and interface modification being applied will suppress the mutual diffusion layer [201].

### 3.2. Interfaces Between the SSEs and the Anode

**Figure 16:**
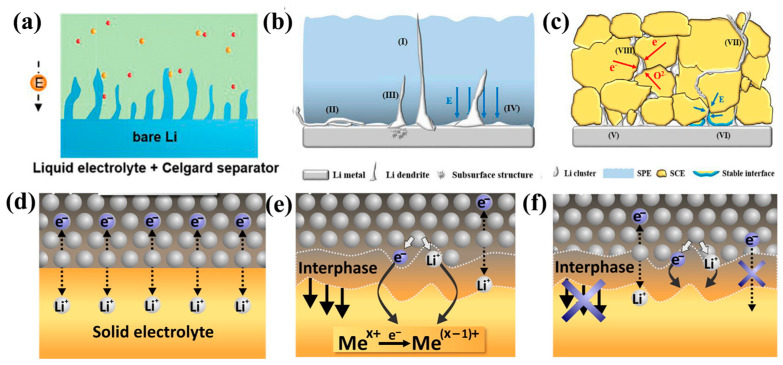
An illustration of Li deposition behaviors: (**a**) liquid electrolyte + Celgard separator [204]; copyright 2019, Wiley; (**b**) organic solid polymer electrolyte; and (**c**) inorganic ceramic/glass electrolyte [205], copyright 2020, Cell Press. (**d**–**f**) Types of interfaces between Li metal anode and solid electrolytes [120], copyright 2022, Wiley.

The use of a lithium metal anode can improve the energy density of lithium batteries, due to the resultant high theoretical specific capacity and a low electrochemical window. During the charging and discharging process of traditional lithium batteries, the uneven deposition of lithium ions on the negative electrode surface can produce lithium dendrites (as shown in Figure 16a), which can puncture the diaphragm and lead to a short circuiting of the battery. Solid-state electrolytes are expected to enhance the energy density of lithium batteries and inhibit the growth of lithium dendrites. A solid electrolyte with a high modulus, a high mechanical strength, and a high density can prevent lithium dendrites from penetrating the diaphragm and causing short circuits. Unfortunately, due to the gap between the lithium metal and the solid-state electrolyte interface, lithium ions cannot be uniformly deposited on the negative surface, leading to the formation of lithium dendrites. For example, in regard to inorganic solid electrolytes, lithium-ion deposits at the inner grain boundary of the electrolyte will also lead to the growth of lithium dendrites (Figure 16b,c). A reduction in SSEs due to lithium metal results in the creation of an interphase between the SSEs and lithium metal anodes. Based on previous experimental and theoretical studies, this interphase can be divided into three types. First, denotes situations where there are no decomposition reactions at the interface between the SSE and the lithium anode (Figure 16d). Second, denotes a situation where the SSE reaction with Li forms a mixed conductive phase (MCI), where ions and electrons are transported at the interface simultaneously (Figure 16e). The interfacial decomposition reaction cannot be terminated, eventually resulting in a short circuit of the cell. Third, denotes a situation where a stable passivation layer gradually forms at the interface as the battery cycle progresses, thus terminating the interfacial reaction (Figure 16f) [203]. Although poor electrode/electrolyte interfacial contact, the electronic conductivity of bulk SSEs, and grain boundaries are supposed to be the cause of dendrite growth in SSEs [100], the mechanism for Li dendrite formation in SSEs is still not fully understood.

There exists an interface instability factor at the interface between the anode and solid-state electrolytes in all-solid-state lithium batteries, except for the compatibility/wettability of the interface and the growth of lithium dendrites. Solid polymer electrolytes with high chemical reactivity exhibit instability at the interface of lithium metal electrodes. For example, PAN has a high electrochemical stability window (4.5V vs. Li/Li^+^), which is believed to match the high-voltage positive electrode. However, the -CN group will react with the lithium metal at the interface to form a passivation layer, which will seriously weaken the performance of the battery.

**Figure 17:**
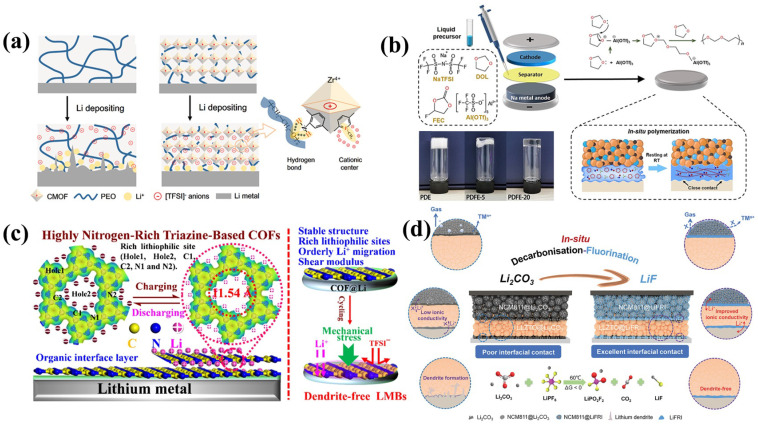
Schematic diagram of a method for inhibiting the growth of lithium dendrites: (**a**) PEO(LiTFSI) electrolyte and anion-immobilized P@CMOF electrolyte [207]; copyright 2019, Elsevier. (**b**) Illustration of the in situ polymerization preparation of the poly(DOL)-based polymer electrolyte [208]; copyright 2023, Elsevier. (**c**) In situ polymerization of triazine COF on lithium metal surface [209]; copyright 2023, Royal Society of Chemistry. (**d**) In situ conversion of the surface Li_2_CO_3_ to LiF-rich interfaces (LiFRIs) on all the surfaces across the cathode and LLZTO [210]; copyright 2023, Wiley.

Numerous methods have been employed to construct stable Li-metal anode/SSE interfaces to address the interfacial issues associated with Li-metal dendrite formation and interfacial reactions. Inorganic fillers possess the capability to provide mechanical support and impede dendrite penetration [179]. A thin and uniform SSE interphase layer can also be formed on the surface of lithium metal using an in situ method [189]. For instance, an artificial SSE interphase film can be affixed to the surface of the Li anode, thereby enhancing the stability of the Li-metal anode and delaying the emergence of Li dendrites and other parasitic interactions between hybrid electrolytes and the Li anode [196]. The design of a 3D composite lithium anode can effectively disperse the lithium ions at the interface between the solid electrolytes, which can reduce the growth of dendrites. The flexible interface layer, which can accommodate variations in the 3D composite lithium anode throughout the cycle, can ensure continuous anode and electrolyte interaction. Lithium anodes may benefit from an electrolyte composed of a polymer matrix and inorganic fillers that trap anions so that lithium ions can be uniformly deposited on the surface of the lithium metal, which will inhibit the growth of lithium dendrites. The flexible interface layer between the composite electrolyte and the lithium anode can cause the lithium anode to adhere to the surface of the lithium anode without any volume change during circulation, improving the uniformity of the flow of lithium ions at the interface. In addition, a multilayer solid electrolyte was designed to maintain the ionic conductivity of the electrolyte and suppress the penetration of Li dendrites at the same time. The multilayer electrolyte includes a interlayer with poor stability and double layer with good stability on both sides to contact the lithium metal surface [206]. As shown in Figure 17, there are several methods for inhibiting the growth of lithium dendrites. A lithium metal composite anode was prepared by optimizing the structure or composition of the lithium metal anode, which exhibited high stability in regard to SEs and can effectively impede the growth of lithium dendrites. Sun et al. [207] have proposed a novel cationic metal–organic framework (CMOF) that could immobilize anions and facilitate the uniform distribution of Li^+^, thereby enabling the construction of dendrite-free SSBs (Figure 17a). Xiang et al. [208] reported on the in situ polymerization of poly(1,3-dioxolane) (DOL), with a volume ratio of 20% of fluoroethylene carbonate (FEC), to enhance the mechanical congruence of electrolyte–electrode interfaces, resulting in the formation of a stable NaF-rich solid electrolyte interphase (SEI) at the anode (Figure 17b). Li et al. [209] synthesized covalent organic frameworks (COFs) with a high Young’s modulus (3.51 GPa) and lots of lithiophilic sites on the Li-metal surface to reduce side reactions, making the Li^+^ flux uniform and promoting Li plating/stripping and suppressing dendrite growth (Figure 17c). Yan et al. [210] employed a decarbonization–fluorination strategy to effectively suppress parasitic reactions and substantially reduce interface resistance, producing a dendrite-free Li anode (Figure 17d). Lithium alloy anodes (such as Li-In [211], Li-Mg [212], Li-Sr [213], and Li-Sn [214]) also provide a promising option for achieving better cyclic performance by stabilizing the SE/anode interface [215]. Due to the high lithium diffusion coefficient of alloy cathodes, the lithium atoms are spread across the anode surface to achieve uniform lithium deposition and avoid the formation of lithium dendrites [216]. Furthermore, the reduction potential of SSEs is higher than that of lithium, which may inhibit the electrochemical decomposition of SSEs. Unfortunately, the expansion of some alloy materials during the battery cycle can limit their further application [217]. Meng et al. [218] devised an in situ preparation involving a 10 nm thin film of a covalent organic framework (COF) uniformly coated on a Li anode (COF-Li) and utilized as an artificial SEI layer, for the purpose of stabilizing the Li plating/striping and inhibiting Li dendrite formation. Yu et al. [219] summarized the structural design of a flexible solid-state lithium–sulfur battery to obtain a high-load, flexible sulfur cathode, to inhibit the growth of lithium dendrites and to achieve flexible battery packaging, with a small sacrifice in terms of energy density.

### 3.3. Interfaces Between the SSEs and the Cathode

In regard to the cathode/electrolyte interface, in general, a large contact area between the cathode and the electrolyte, a stable interface, and enough ion transport channels, are necessary for a lithium battery to benefit from high capacity and energy density. The main interface issue relates to the cathode material during the long-term charge and discharge process, due to the volume changes resulting from the constant contractions and expansions that lead to inadequate contact between the electrode and the electrolytes. Moreover, the instability of the cathode/electrolyte interface and the adverse oxidation side reactions caused by electrochemical incompatibility can eventually induce the aging/evolution of the interface during the long-term cycles of the battery. To improve the energy density of lithium batteries, high-voltage cathode materials are used. Therefore, solid electrolyte materials with high electrochemical windows are required. However, the main difference between solid electrolytes and liquid electrolytes is that the interface between the cathode and the solid electrolyte lacks the wettability and tight physical contact needed to produce high interface resistance [220]. Meanwhile, the chemical and electrochemical reactions occurring between the cathode and the SSEs and the element diffusion at the interface lead to electrolyte degradation when subject to a high electrochemical oxidation window and the decay of the electrochemical performance of the battery.

**Figure 18:**
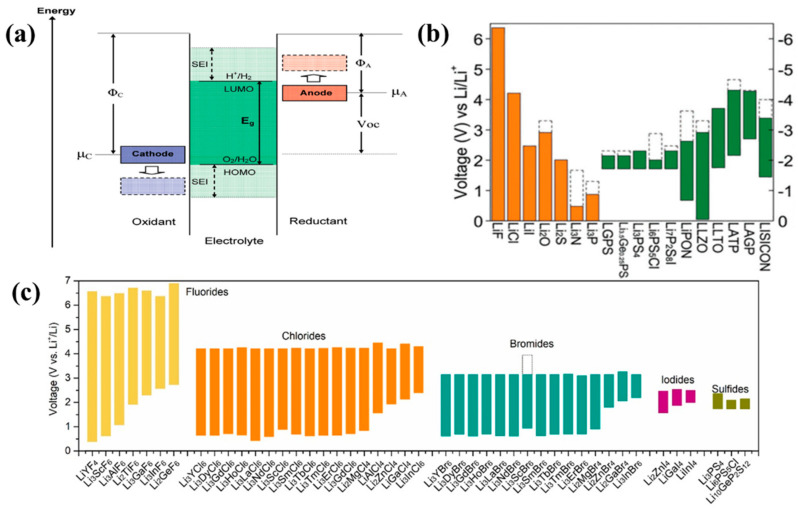
(**a**) ΦA and ΦC are the anode and cathode work functions. Eg is the window of the electrolyte in regard to its thermodynamic stability. μ_A_ > LUMO and/or a μ_C_ < HOMO requires a kinetic stability related to the formation of an SEI layer [221]; copyright 2010, American Chemical Society. (**b**) Solid color bars represent the electrochemical stability windows of SEs and some lithium compounds. The oxidation potential to fully dilatate these materials is marked by the dashed line [223]; copyright 2019, American Chemical Society. (**c**) The electrochemical window of halide SSEs in comparison with sulfides [153]; copyright 2022, Science.

SSEs with a narrow electrochemical window are prone to being oxidized when in contact with oxide cathodes at high voltages. The reaction at the electrode/electrolyte interface can be categorized into three different scenarios, depending on the conditions occurring at the time. First, the SSEs are reduced or oxidized at an applied potential beyond the electrochemical window. Second, a chemical reaction will occur if the chemical potential of the SSEs and the electrode do not match. Third, the electrochemical reaction between the electrolyte and the electrode takes place during the charging and discharging process of the solid-state lithium battery [120]. The electrochemical stability of SSEs is controlled by the electrochemical stability window, which is related to the highest occupied molecular orbital (HOMO) and the lowest unoccupied molecular orbital (LUMO) of the electrolytes (Figure 18a) [221]. The electrochemical stability window is very important for the interface stability and will reflect the interface compatibility between the solid electrolyte and the cathode material. Density functional theory (DFT) calculations are a useful tool to predict the electrochemical stability window. Mo et al. [222] calculated the electrochemical windows of various inorganic SSEs using first-principles calculations and density functional theory (Figure 18b). As shown in Figure 18c, Sun et al. [153] studied the voltage stability of halide and sulfide SSEs to demonstrate that halide SSEs have the widest electrochemical window, that is, high oxidation stability and low reduction stability.

Polymer electrolytes, such as PEO-based electrolytes, exhibit low ionic conductivity, insufficient thermal stability, limited oxidation stability (<3.8 V), and insufficient mechanical strength, and which are not stable with high-voltage cathodes (>4.0 V). Sulfide electrolytes have a narrow electrochemical stability window, being incompatible with high-pressure cathode materials (>4 V), and an interface side reaction will take place with positive electrode materials [224,225]. Compared to sulfide SES, most oxide SES have a wider range of electrochemical stability and a higher stability with positive electrode materials. However, high-temperature sintering can achieve good interface contact between the positive electrode material and hard oxide SEs. In the meantime, high-temperature sintering will cause chemical reactions at the interface, leading to interface instability [85].

**Figure 19:**
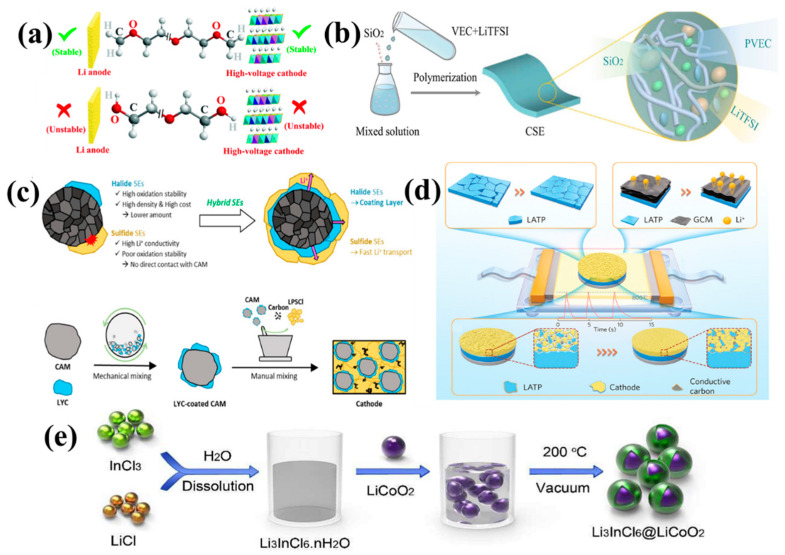
The various strategies to deal with side reactions at the interface and improve the electrochemical oxidation window of solid electrolytes: (**a**) PEGDME and PEG response to the Li anode and a high voltage [226]; copyright 2020, Royal Society of Chemistry. (**b**) Schematic diagram of preparing the CSE membrane from a VEC precursor with Li salt and nanoparticles [227]; copyright 2022, Elsevier. (**c**) The advantages and disadvantages of halide and sulfide cathodes and the corresponding diagram of halide–sulfide mixed electrolyte-coated cathodes [230]; copyright 2023, Elsevier. (**d**) Illustration of that the cathode/SES enhances the density of SSE at a ultrahigh heating rate by filling the void and obtains good cycle stability [234]; copyright 2023, Wiley. (**e**) Illustration of the in situ synthesis of Li_3_InCl_6_ on LiCoO_2_ (LIC@LCO) [236]; copyright 2020, Elsevier.

Researchers have adopted various strategies to deal with side reactions at the interface and improve the electrochemical oxidation window of solid electrolytes to solve the problem at the interface between solid electrolytes and positive electrode materials (as shown in Figure 19). The introduction of a passivation layer into the solid electrolyte will avoid direct contact with the positive electrode material and it will reduce the surface catalysis of the positive electrode material and the side reaction between the solid electrolyte and the positive electrode. Yang et al. [226] have discovered that the stability window can be extended from 4.05 to 4.3 V (Figure 19a) by replacing -OH with more stable -OCH_3_. Sun et al. [207] synthesized a novel cationic metal–organic framework (CMOF) by grafting with the -NH_2_ group to protect the ether oxygen of polymer chains through hydrogen bonds, thereby extending the electrochemical window to 4.97 V. Yu et al. [227] reported that a battery with the LiCoO_2_ cathode presented good cycle stability at a high voltage by incorporating SiO_2_ nanoparticles into the poly(vinyl ethylene carbonate) polymer electrolyte (Figure 19b). Zhang et al. [228] designed a flexible anion-immobilized ceramic–polymer composite electrolyte, in which the ceramic fillers in the electrolyte extended the electrochemical stability during a wide voltage range from 0 to 5.5 V (vs. Li^+^/Li). Wang et al. [229] found that the interfacial side reactions between NCM and solid polymer electrolytes can be mitigated by constructing an interfacial nanolayer with aromatic polyamide (APA) on the surface of NCM active particles (NCM@APA). An electrochemical window stability of 4.3 V was achieved. Jung et al. [230] further improved the electrochemical performance by coating a mixed-conducting phase comprising LYC and carbon on Li[Ni_0.88_Co_0.11_Al_0.01_]O_2_ (NCA) particles (Figure 19c). Furthermore, the introduction of a coating between the solid electrolyte and the oxide cathode weakens the space-charge effect, thereby preventing adverse interface reactions. The coatings include lithium-ion conductive coatings (such as LiNbO_3_ [231], Li4Ti_5_O_12_ [232], and Li_2_SiO_3_ [233]) and dielectric oxides (Al_2_O_3_ [166]), both of which significantly reduce the cathode interface resistance. These coating materials have a wider electrochemical window, which improves the stability of the electrode/electrolyte interface during cycling. Sun et al. [234] reported the fabrication of LATP-based solid-state batteries through innovative thermal pulse sintering (TPS), which made significant progress by delivering a favorable cycle stability at 4.6 V with a LiCoO_2_ cathode (Figure 19d). Duan et al. [235] proposed a heterogeneous multilayered solid electrolyte to broaden the electrochemical window of solid electrolytes to 0–5 V, which would overcome the interfacial instability problems that hinder high-voltage solid-state Li metal batteries. Sun et al. [236] successfully addressed the long-standing interfacial difficulties by establishing the in situ interfacial growth of a highly Li^+^-conductive halide electrolyte (Li_3_InCl_6_, LIC) on the cathode’s surface (Figure 19e).

## 4. Industrialization Status of Solid-State Electrolytes

The application of solid-state batteries will lay the foundation for the upgrading of the lithium battery industry, due to their high-energy density and superior safety. In particular, solid-state batteries with a high-nickel ternary positive electrode and a metal lithium negative electrode material can possess an energy density of up to 400 Wh/kg, far more than liquid lithium-ion batteries. Such a high-energy density can greatly extend the driving range of electric vehicles, eliminate consumer concerns, expand market demand, and greatly improve the safety of lithium batteries. At present, global solid-state battery research and development can be divided into three camps, namely research taking place in Japan and South Korea, Europe and the United States, and China. The sulfide solid electrolyte technology route has been selected by Japan and South Korea. Toyota, the world’s leading manufacturer of solid-state batteries, has applied for numerous patents on solid electrolytes. European countries and America have selected the oxide solid electrolyte technology route, which has a wide range of layouts and directly involves the development of the application of lithium metal anodes. The research demonstrates a significant increase in the energy content of lithium batteries. China has been involved in the development of all-solid-state batteries and is also putting a lot of effort into developing semi-solid batteries to fit in and satisfy the existing requirements of the industry. The domestic solid-state battery industry involves traditional battery operations, downstream raw material markets, and specialized solid-state battery companies, and there are numerous technical routes to choose from, but there is still a lag in comparison to the global leader in this technology.

At present, solid-state batteries have made some progress in terms of mass production and application. Ningde Times has released condensed matter batteries, with a single energy density of 500 Wh/kg, which are expected to be applied to civil electric manned aircraft projects in 2023. The Qingtao Energy Joint SAIC has established a joint laboratory for solid-state batteries and will cooperate on solid-state battery projects. The 360 KWh/kg semi-solid-state battery created by Weilai New Energy was officially handed over to the NIO. The semi-solid-state battery loading test is being promoted by both domestic and foreign car manufacturers to enhance their competitiveness, including Toyota, Nissan Honda, Dongfeng, Beiqi, Blue Valley, Volkswagen, BMW, and others. In other words, solid-state batteries are a promising technology as both technological advancements and improvements will continue. The development prospects for solid-state batteries will broaden and the development of solid-state battery technology will be divided into three stages: the semi-solid state, the quasi-solid state, and the all-solid state. For all-solid-state batteries, there are still some technical issues, such as low ionic conductivity, high interface impedance, and poor cycle performance. The need for a further breakthrough in regard to semi-solid-state batteries could involve the addition of a small amount of electrolytes based on the all-solid-state battery to improve the interface performance of the electrolytes and the electrode. At present, the semi-solid state is the current more mature technical route. The key performance of solid-state batteries is determined by solid-state electrolytes. At present, the main types of solid-state electrolytes studied in regard to industrialization are polymers, oxides, sulfides, and halide electrolytes. Polymer electrolytes have the advantages of high conductivity at high temperatures, easy processing interface impedance control, etc., and they represent the earliest technical route to industrialization. Oxide electrolytes have high conductivity at room temperature, good electrochemical stability, good cycling performance, and other advantages, but the contact at the interface is weak, leading to a high level of interface impedance. Sulfide electrolytes have the highest conductivity at room temperature, unstable interface stability, and good oxidation properties. The positive and negative electrode materials used in solid-state batteries are roughly the same as those in traditional lithium-ion batteries, mainly graphite or silicon–carbon materials in the negative electrodes and composite materials in the positive electrodes. Only solid electrolytes are used to replace traditional organic electrolytes and the diaphragm; solid-state batteries can also be developed into solid-state lithium metal batteries, that is, lithium metal is used as the negative electrode, or non-lithium metal anode, and can also use sulfur-containing materials or lithium–air batteries to select and optimize the positive and negative materials in solid-state batteries. Halide electrolytes are currently attracting the attention of scientific researchers because of their high ionic conductivity and high electrochemical oxidation window. The types and development status of the solid electrolytes currently being studied by some countries and enterprises are listed in Table 5 [94].

## 5. Conclusions and Future Perspectives

With the continuous demand for electric vehicles and electronic devices, the pursuit of energy storage devices that offer superior safety and energy density has accelerated the development of solid-state lithium batteries. Certain electrolyte materials with high ionic conductivity have progressed to the industrialization stage, but the progression to the application of solid-state batteries from the basic research carried out in the laboratory to the industrialization process is still faced with numerous obstacles and challenges. The research progress on solid electrolytes and the migration mechanism of lithium ions are discussed in this review. The interface issues involving the contact between solid electrolytes and lithium metal anodes and cathodes are also discussed, such as the high interface impedance in regard to electrode materials, side reactions involving electrodes, the growth of lithium dendrites, and the breakdown of electrolyte materials at a high voltage. Based on this foundation, efficient optimization strategies for reducing electrode/electrolyte interface impedance, enhancing interface stability, and inhibiting lithium dendrites are summarized, including electrode coating, electrolyte composition adjustment, and interface construction. The industrialization research status of solid-state batteries and solid-state electrolytes in different countries are introduced, especially in China.

At present, solid-state lithium batteries are not able to meet the practical application and commercialization requirements, and there are still many problems to be solved. The potential development directions and prospects for high-energy density solid-state batteries can be summarized as follows:

(1) Material and structural innovations related to solid electrolytes: There is still a significant gap in the ionic conductivity between solid electrolytes and traditional liquid electrolytes. It is important to optimize the ionic conductivity of solid electrolytes. New solid electrolyte materials, such as ceramics, polymers, and gels, etc., are being developed through material innovation, structural design, and computer-aided functions to improve the conductivity and stability of materials at the interface. The battery’s mechanical and electrochemical properties will be enhanced by innovative solid electrolyte structures, such as gradient structures, composite structures, and sandwich structures. For example, at the microscopic scale, the crystal structure of fast ionic conductors can be enhanced by improvements to the preparation process or material composition. At the macro level, the density of the electrolyte layer can be improved by incorporating additional additives or enhancing the processing parameters of the material to attain a higher level of ionic conductivity;
(2) Optimization of the interface stability of solid-state battery electrodes and reducing interface impedance: The battery’s electrochemical stability and cycle duration can be promoted by enhancing the contact area between the electrode and solid electrolytes through surface coating treatment and element doping. Moreover, the oxidation stability of SSEs and controlling the growth of lithium dendrites can be improved by optimizing the preparation process and the material composition of solid electrolytes and the construction of an artificial in situ SEI layer. The contact properties of materials can be effectively improved by designing matching single-crystal or polycrystal materials and solid-state battery processes. Likewise, new preparation procedures are being developed, such as 3D printing, solution casting, vapor deposition, in situ polymerization, dry mixing, and other procedures to decrease the interface resistance of solid electrolytes. In the future, the development of new binders, fillers, and other additives will also make a significant contribution to solving the problems related to poor interface contact and high interface resistance;
(3) The design of inorganic/polymer composite solid electrolytes with new structures: The electrolytes of inorganic/polymer composites have good mechanical workability, ion conductivity, and electrochemical stability, being one of the best choices for incorporation within the all-solid-state battery system in the future, to realize the complementary advantages of polymer electrolytes and inorganic electrolytes, and to establish a rapid ion transport channel between the interfaces in each phase of the composite electrolyte;
(4) Evaluation techniques for testing solid-state batteries and solid electrolytes and the characterization of advanced technology: Establish a complete solid-state battery test and a solid-state electrolyte test evaluation system, including electrochemical performance tests and safety performance tests, etc., to accelerate the commercialization of solid-state electrolytes and solid-state battery industrialization processes. The utilization of in situ Raman technology, in situ Fourier infrared technology, hashes, and other technologies can be used to assist the fundamental theoretical research into solid electrolytes, including the transmission mechanism of the solid electrolyte interface, to guide the development of novel materials and novel structures, and expedite the development of solid lithium batteries.

Solid-state battery research and industrialization still have a long way to go. The development of all-solid-state lithium batteries needs to be considered from the perspective of the entire battery system. It is essential that basic laboratory research and industrialization research work closely together to promote the rapid development of all-solid-state lithium batteries.

## Figures and Tables

**Table 2:** The ionic conductivity, advantages, and disadvantages of different types of inorganic solid electrolytes.

Type	Conductivity (S cm^−1^)	Advantages	Disadvantages	Refs.
Oxide electrolytes	10^−5^–10^−3^	High chemical/mechanical stability High electrochemical oxidation voltage (0–5 V)	Rigid structure High processing temperature High interfacial resistance at the electrode–electrolyte interface	[13,70,71]
Sulfide electrolytes	10^−7^–10^−2^	Excellent conductivity Flexible shape High mechanical strength	Low oxidation stability Sensitivity to moisture Low compatibility with cathode materials	[72,73]
Li–halides electrolytes	10^−8^–10^−3^	Stable with lithium metal High mechanical strength Electrochemical stability	Instability in regard to cathode materials Low ionic conductivity	[74,75]

**Table 3:** The Li^+^ conductivity and activation energy of halide solid electrolytes at room temperature (RT).

Halide SEs	σLi (mS cm^−1^) @RT	Ea	Refs.
Li_3_InCl_6_	1.49–2.04	0.35 eV	[145]
Li_2_ZrCl_6_	0.446	0.31 eV	[146]
Li_2.25_Zr_0.75_Fe_0.25_Cl_6_	0.98	0.346 eV	[154]
Li_2_In_x_Sc_0.666−x_Cl_4_	1.83–2.03	0.33 eV	[155]
Li_2.73_Ho_1.09_Cl_6_	1.3	0.4 eV	[156]
Li_6_PS_5_Cl_0.25_Br_0.75_	3.9	/	[126]
Li_2.6_Yb_0.6_Hf_0.4_Cl_6_	1.5	0.26 eV	[157]
Li_3_InBr_3_Cl_3_	0.12	0.21 eV	[158]
1.5Li_2_O-TaCl_5_	6.6	0.274 eV	[159]
A-LTC	7.16	/	[160]

**Table 4:** Influence of inorganic fillers and additives on ionic conductivity.

Polymer/Li Salt	Filler	Concentration	Morphology	Additive	t_Li+_	σ (S cm^−1^)	Refs.
PEO-PVDF/LiNO_3_	Al_2_O_3_	2%	Nanoparticle	/	0.33	1.25 × 10^−4^/30 °C	[166]
PEO/LiClO_4_	SiO_2_	10%	Nanoparticle	/	/	4.4 × 10^−5^/30 °C	[179]
PEO/LiTFSI	Li_6.4_La_3_Zr_2_Al_0.2_O_12_	/	3D	/	/	2.5 × 10^−4^/RT	[180]
PAN/LiClO_4_	LLTO	15%	Nanowires	/	/	2.4 × 10^−4^/RT	[181]
PEG-PEO/LiTFSI	LGPS	3%	Micro-sized	CTMS	0.68	9.83 × 10^−4^/RT	[182]
PVA/LiCF_3_SO_3_	PDA-coated LLZTO	7%	Nanoparticles	60%BMIMOTf	0.76	2 × 10^−3^/RT	[183]
PEO	LLZO	/	/	SN	0.35	0.74 × 10^−4^/RT	[184]
PEGMEMA/LiTFSI	LAGP	25%	Nanoscale	AIBN	0.87	2.37 × 10^−4^/RT	[185]
PEO/LiTFSI	Li_6_PS_5_Cl	10%	Micro-sized	Ionic liquid	/	2.47 × 10^−4^/25 °C	[186]
PEO-PVDF	Li_0.33_La_0.55_TiO_3_	8%	Nanowires	DMF + glycerin	0.86	6.02 × 10^−3^/25 °C	[187]

**Table 5:** Research status of solid-state electrolytes in some countries and by some enterprises.

Country	Corporation	Solid-State Electrolyte Type	Present Situation
China	Welion New Energy	Hybrid oxide solid–liquid electrolyte	Achieves an energy density of 360 Wh kg^−1^, was mass produced in 2022, can power an EV for 1000 km on a single charge.
	ProLogium	Hybrid oxide solid–liquid electrolyte	Achieves energy densities of 383 Wh kg^−1^ and 1025 Wh/L for 500 cycles. The company plans to start trial production of the ASSLBs in 2023 and mass production in 2024.
	QingTao	Hybrid oxide solid–liquid electrolyte	Achieves an energy density of 368 Wh kg^−1^ and a discharge capacity (1/3C) of over 116 A.
	Ganfeng Lithium	Hybrid solid–liquid/solid electrolyte	Second-generation ASSLBs, with an energy density of 360 Wh kg^−1^ are under investigation.
	Gotion High-tech	Hybrid solid–liquid electrolyte	Achieves a capacity of 136 Ah and an energy density of 360 Wh kg^−1^.
	CATL	Sulfide SE	The ASSLBs are expected to be commercialized around 2030.
	SUNWODA	Sulfide SE	Among them, the latest ampere-hour samples of the first-generation of all-solid-state batteries can achieve a stable cycle of more than 1000 weeks. The second-generation laboratory samples have reached an energy density target of 500 Wh/kg.
	SVOLT	Sulfide SE	The company created in July 2022 a 20 Ah-class ASSLB that can reach an energy density of 350 Wh kg^−1^.
	Enpower	Sulfide SE	Developed a prototype ASSLB, which has a capacity retention of 80% after 1000 cycles at 1C and 100% depth of discharge (DOD).
	Mache Power	Sulfide SE	Achieves an energy density of 250 Wh kg^−1^.
	High Energy Era	Sulfide SE	Prepared 1.46 Ah sulfide-based ASSLB, with an energy density of >330 Wh kg^−1^.
	China Automotive Innovation Corporation	Sulfide SE	The trial production of 10 Ah ASSLBs has been successfully completed.
USA	Solid power	Sulfide SE	0.2 Ah Li metal-anode ASSLBs have been successfully assembled and are under evaluation.
	Quantumscape	Oxide SE	Their batteries have achieved 1000 Wh/L, 350 Wh kg^−1^, and 4C fast charge.
	SES	Hybrid solid–liquid electrolyte	Released a 107 Ah battery, Li-metal battery worldwide, with 935 Wh/L and 417 Wh kg^−1^.
	Factorial Energy	Factorial electrolyte	The 40 Ah SSLB demonstrated a 97.3% capacity retention rate after 675 cycles.
Japan	Toyota	Sulfide SE	In September 2020, an all-solid-state battery prototype vehicle was built and driving data were obtained.
	Hitachi Zosen	Sulfide SE	The company announced that it has developed an ASSLB with a capacity of 1 Ah and capable of operating at harsh temperatures (−40–120 °C).
	Nissan	Sulfide SE	The pilot plant in Yokohama will be ready by 2024 and CNY 140 billion will be invested into the ASSLB by 2026.
South Korea	Samsung SDI	Sulfide SE	In March 2020, Samsung SDI released a prototype pouch ASSLB with a Ag-C anode, which achieved a high-energy density (>900 Wh/L) and a long cycle life (1000 cycles).
	LG Chem	Sulfide/polymer SE	Achieved an ASSLB with an all-silicon anode, which reached a capacity retention of 80% after 500 cycles.
	SK Innovation	Sulfide SE	SK Innovation chose Solid Power as a partner and has invested USD 30 million to develop SSLBs, with an energy density of 930 Wh/L.
Europe	Oxis Energy	Hybrid solid–liquid electrolyte	OXIS has filed nine new families of patents to protect both quasi- and solid-state intellectual property rights.
	Ilika	Oxide SE	SSLBs have demonstrated >500 cycles without failure and 1C discharge cycling at 25 °C.
	Bollore	Polymer PEO	Achieved an energy density of >250 Wh kg^−1^ and cycling life of over 4000 times at 50–80 °C.

## Data Availability

No data were used during the research described in this article.
